# Preliminary effectiveness and implementation outcomes of the IMARA-South Africa sexual health intervention on adolescent girls and young women: A pilot randomized trial

**DOI:** 10.1371/journal.pgph.0001092

**Published:** 2023-02-15

**Authors:** Katherine G. Merrill, Millicent Atujuna, Erin Emerson, Dara Blachman-Demner, Bethany C. Bray, Linda-Gail Bekker, Geri R. Donenberg

**Affiliations:** 1 Department of Medicine, Center for Dissemination and Implementation Science, University of Illinois Chicago, Chicago, IL United States of America; 2 Desmond Tutu HIV Centre, University of Cape Town, Cape Town, South Africa; 3 Office of Behavioural and Social Sciences, National Institutes of Health, Bethesda, MD, United States of America; 4 Institute for Health Research and Policy, University of Illinois Chicago, Chicago, IL, United States of America; University of the Witwatersrand, SOUTH AFRICA

## Abstract

Family-based interventions may help reduce the risk of HIV and other sexually transmitted infections (STI) among adolescent girls and young women (AGYW) in sub-Saharan Africa but few have been tested. We examined the preliminary effectiveness and implementation outcomes of *I**nformed*, *M**otivated*, *A**ware*, *and* *R**esponsible* *A**dolescents and Adults—**S**outh* *A**frica (IMARA-SA)*, an evidence-based intervention for South African AGYW (15–19 years) and their female caregivers. We piloted IMARA-SA in the Western Cape using an individually randomized experimental design and average follow-up at 11 months. Primary outcomes were HIV Testing and Counselling (HTC) uptake, STI incidence (gonorrhea, chlamydia), and pre-exposure prophylaxis (PrEP) uptake. Secondary outcomes were self-reported sexual risk behavior (condom use at last sex, consistency of condom use, substance use during sex, and number of sexual partners) and PrEP adherence. We examined four implementation outcomes: reach, feasibility, acceptability, and fidelity. Data from 59 AGYW (mean = 17.2 years) were analyzed at baseline (n = 29 from IMARA-SA, 30 from a health promotion control group). At follow-up, 51 (86%) completed surveys and 39 (66%) presented for HTC, STI testing, and/or PrEP. Compared to controls, fewer IMARA-SA participants tested positive for an STI (22% versus 38%), more IMARA-SA participants took up PrEP (68% versus 45%), and four of five secondary outcomes favored the IMARA-SA group at follow-up. These differences did not reach statistical significance. HTC uptake at follow-up was 100% in both groups. All AGYW-FC dyads agreed to participate in the study (reach). In the IMARA-SA group, 76% of dyads completed the intervention (feasibility), and over 76% of acceptability ratings from AGYW and their FC had the highest Likert rating. Fidelity of intervention delivery was 95%. IMARA-SA is a promising strategy for reducing HIV/STI risk among South African AGYW. We found strong evidence of reach, feasibility, acceptability, and fidelity. A fully powered randomized controlled trial is warranted.

**Trial registration: Clinical trials.gov registration number:** NCT05504954.

## Introduction

Despite progress achieved in reducing HIV infections in sub-Saharan Africa [1], adolescent girls and young women (AGYW) remain disproportionately impacted. AGYW aged 15–19 years comprise 80% of new HIV infections among adolescents in the region. In South Africa, which has the highest number of people living with HIV globally [2], more than twice as many young women are living with HIV (10.2%) than young men (3.4%) [3]. Structural inequities, including relationship power differentials, gender-based violence, and economic vulnerability, exacerbate the challenges South African AGYW face in protecting themselves from HIV [4].

Studies demonstrate the important role that families—in particular, female caregivers (FC)—play in sexual health decision-making among AGYW in sub-Saharan Africa. AGYW typically prefer talking to their mothers about sexuality issues [5, 6], despite taboos which make these conversations uncomfortable [5]. AGYW are more likely to postpone sex and use condoms after sexual debut if they have a parent/caregiver who monitors their activities and can communicate openly about sexual health issues [7, 8]. However, few family-focused HIV programs have been tested in sub-Saharan Africa [9, 10], and those programs that do exist have generally targeted younger adolescents (aged 10–14 years) [11] or youth living with HIV [12] rather than AGYW.

*I**nformed*, *M**otivated*, *A**ware*, *and* *R**esponsible* *A**dolescents and Adults* (IMARA) is an evidence-based mother-daughter HIV/sexually transmitted infection (STI) prevention program (“mother” referring to any female caregiver) originating in the United States. It was designed for African American families using the social-personal framework [13] and blends three evidence-based interventions [14–17]. An efficacy trial in Chicago found that girls (aged 14–18 years) who received IMARA were 43% less likely to contract a STI during one year of follow-up compared to girls receiving a health promotion program (p = 0.011) [18]. Using the ADAPT-ITT model [19], our team rigorously adapted the intervention for South African AGYW and their FC (e.g. aunts, sisters) over seven months (April-October 2019). Results from the adaptation process, which involved obtaining feedback from the Desmond Tutu Health Foundation’s adult and youth community advisory boards and theater testing with AGYW and their FC, are reported elsewhere [20, 21].

This paper presents findings from a pilot randomized trial of the IMARA intervention adapted for a South African (SA) setting (“IMARA-SA”). We examined its preliminary effectiveness, hypothesizing that we would observe greater HIV testing and counselling (HTC) and pre-exposure prophylaxis (PrEP) uptake, fewer STIs, reduced sexual risk behavior, and better PrEP adherence among IMARA-SA AGYW compared to controls. We also examined four implementation outcomes: reach, feasibility, acceptability, and fidelity. We drew on the RE-AIM framework to look at reach, which offers important insight into those individuals who are willing to participate in an intervention [22]. The remaining three outcomes were selected from Proctor et al.’s Implementation Outcomes Framework (IOF) and are considered “necessary preconditions” for obtaining desired effectiveness outcomes [23].

## Methods

### Study design, setting, and participants

**Fig 1:**
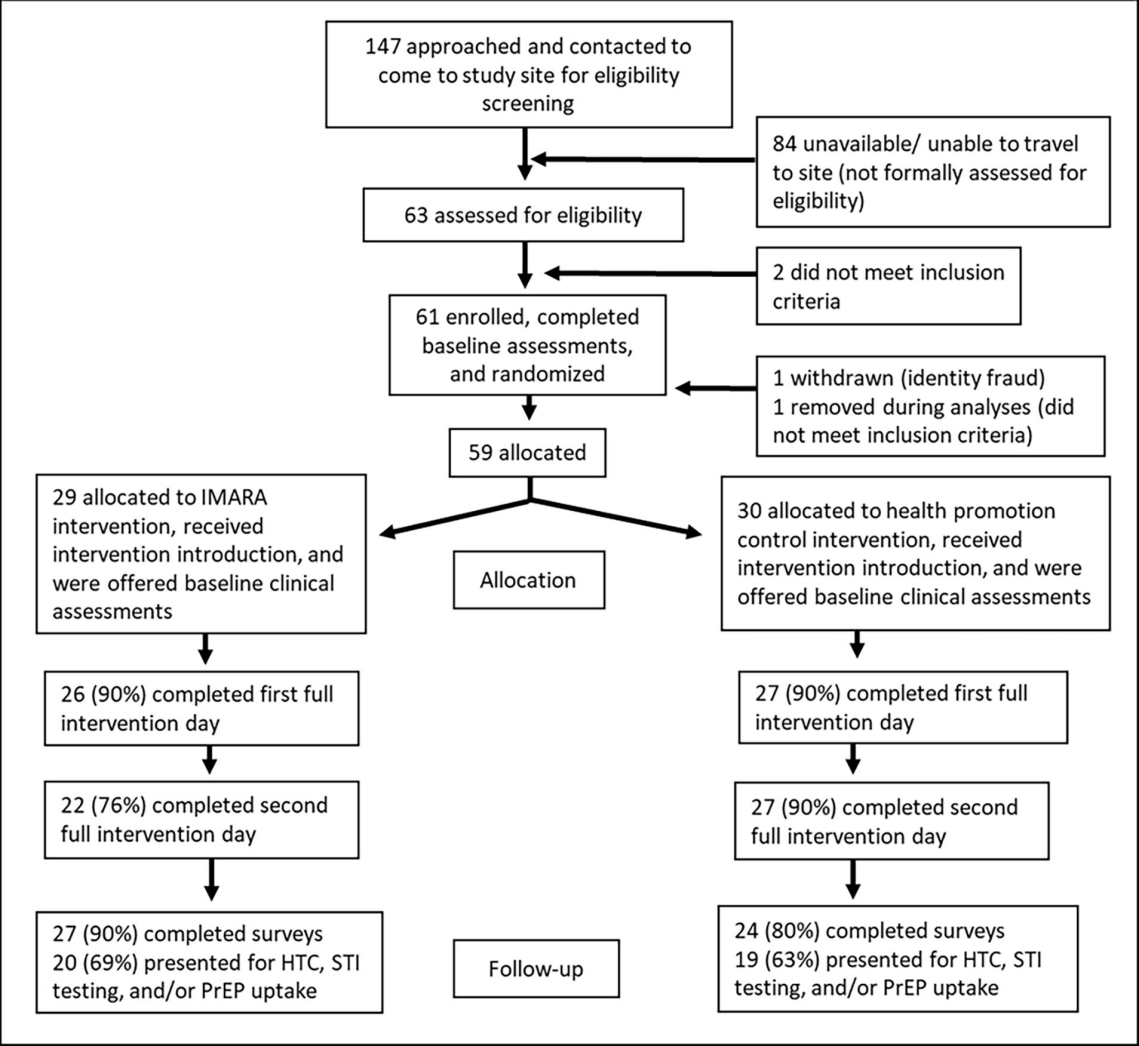
CONSORT diagram. Presents the flow of participants through the pilot study.

In this two-arm pilot project, AGYW-FC dyads were individually randomized to the IMARA-SA or a family-based health promotion control program matched in time and intensity. Baseline and follow-up data collection occurred between October 2019 and May 2021. The study setting was the Klipfontein/Mitchells Plain health sub-district of the Western Cape, which is a densely-populated, low resource, and high disease-burdened region with just over one million residents [24, 25]. AGYW in the area face a high prevalence of HIV (6.7%), pervasive gender-based violence, high STI prevalence, and low condom use [26–28]. Inclusion criteria for AGYW were as follows: female; Black or mixed race (self-identified); 15–19 years-old; residing in Klipfontein/Mitchells Plain or neighboring areas; and English and/or Xhosa speaking. Inclusion criteria for FC were as follows: identified by the AGYW as a FC; 24 years or older; living with or in daily contact with the AGYW; and English and/or Xhosa speaking. AGYW and FC were excluded if they participated in the IMARA-SA adaptation process. AGYW and FC had to agree to participate as a dyad. AGYW refusal superseded FC agreement to participate. Given that this was a pilot study, we chose a sample size that would enable us to assess the feasibility and acceptability of delivering IMARA-SA. Our study was therefore not powered to detect our primary outcomes. Our CONSORT diagram is in Fig 1.

### Procedures

AGYW and FC were approached through street outreach and at community spaces and were given information about the study. Those expressing interest gave their contact details and were scheduled to come to the study site, where study staff conducted screening, consent/assent, and enrolment. During consent, participants indicated their interest in receiving HTC, STI testing (chlamydia, gonorrhea), and/or a PrEP prescription. A 1.5-hour baseline survey was administered using computer-assisted self-interview (CASI), which participants completed independently in English or Xhosa. Surveys asked participants to report on their demographics, sexual risk behavior, PrEP uptake and adherence, and other topics (e.g., mental health [29]). Study staff were available to answer questions. Dyads were randomized by AGYW choosing their program from a paper bag without replacement to ensure equal numbers in each arm. IMARA-SA and control group dyads went to separate rooms for the intervention.

Those who had indicated interest in receiving HTC, STI testing, and/or a PrEP prescription remained at the on-site clinic at the end of Day 1. AGYW testing negative for HIV and opting for PrEP were given a one-month PrEP prescription with referral to a public clinic for refills if cleared to safely use PrEP. HTC results were provided immediately. STI results were given following Day 2 for those testing negative. Those testing positive were called to come to site as soon as STI results were received for risk reduction counselling and treatment.

Participants returned on one or two separate occasions to complete the full intervention. Groups ranged from two to nine dyads. Immediately following each intervention day, participants in both arms completed paper-based surveys about their experience with the intervention. Participants were reimbursed for their time and transport, and snacks and a meal were provided on intervention days.

Observers documented fidelity to the IMARA-SA curriculum on each intervention day. Ratings were reviewed by the SA project coordinator and discussed during supervision meetings. Concerns were addressed by additional training.

Follow-up data collection consisted of surveys and clinical data. AGYW were again offered HTC, STI testing, and/or a PrEP prescription. Due to COVID restrictions and lockdowns, follow-up surveys were conducted using CASI between 6- and 10-months post-baseline (mean = 8.7 months, SD = 0.79) and in-person on-site visits for clinical data collection (HTC, STI testing, and/or a PrEP prescription) took place between 8- and 17-months post-baseline (mean = 13.6 months, SD = 3.7).

### Treatment groups

**Fig 2:**
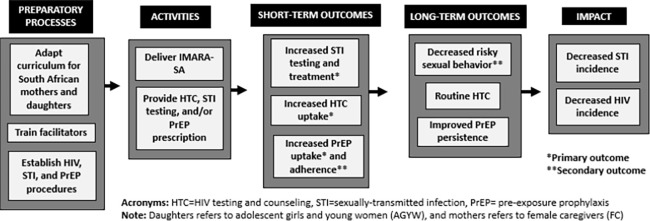
Simplified intervention impact model. Presents how IMARA-SA is expected to lead to the desired impact.

The IMARA-SA intervention integrates separate and joint sessions for AGYW and their FC, which run simultaneously and cover parallel content. Role-playing, skills practice, and interactive activities are emphasized (see S1 Fig for curriculum summary). IMARA-SA’s goals and motto highlight strong AGYW-FC communication and relationships, sisterhood, community empowerment, and motivation for HIV prevention, while building group cohesion. Content aligns with an ecological framework [30] by addressing drivers of HIV-risk at multiple levels. Sessions are designed to take place over two full days (~10 hours). Fig 2 shows a simplified version of our intervention impact model, articulating the pathways between preparatory processes, activities, outcomes, and desired impact for the pilot.

The control group is a time-matched health promotion program, delivered in the same format as IMARA-SA. It is based on a program used in previous research and has no content concerning sexual and reproductive health or strategies to improve AGYW-FC communication in order to distinguish from the key features thought to impact outcomes in IMARA-SA. The curriculum addresses media literacy, types of violence and prevention strategies, negative impacts of substance use, and benefits of healthy eating and physical activity (S1 Fig).

### Facilitator training

Facilitators for both conditions were Xhosa- and/or English-speaking South African women with and without previous experience leading groups. They were trained separately over one month until competency was reached (~30 hours). They did not overlap arms. For both programs, training followed a detailed manual and was led by the US principal investigator and the SA site co-investigator. Emphasis was placed on the importance of manualized interventions, adhering to intervention content, and strategies to facilitate participation. IMARA-SA training reviewed the facts of STI/HIV transmission and prevention, AGYW psychosexual development, group dynamics, and behavior management. Facilitators role-played intervention activities and received feedback. The SA site co-investigator determined competency based on demonstrations of accurate, clear, and comfortable intervention delivery to trainees posing as AGYW and FC.

### Measures

#### Primary effectiveness outcomes

*HTC uptake* was measured as the proportion of AGYW who completed HTC at the on-site clinic at follow-up. *STI incidence* was assessed as the proportion of AGYW testing positive for gonorrhea and/or chlamydia at the on-site clinic at follow-up. *PrEP uptake* was measured as the proportion of AGYW who received a PrEP prescription from the research team (determined by study records) or another care provider after the baseline survey (determined by self-report on the follow-up survey).

#### Secondary effectiveness outcomes

*Sexual risk behavior* was measured using the AIDS-Risk Behavior Assessment (ARBA) [31], a measure of adolescent sexual activity widely used with diverse populations of youth, including adolescents receiving mental health care [32, 33], young men who have sex with men [34], justice-involved teens [35], Rwandan adolescents [36], and African American females [18]. Analyses examined differences at follow-up between intervention groups in four sexual behaviors among AGYW who reported vaginal and/or anal sex in the past six months: condom use at last sex (yes/no); and consistency of condom use (always/less than always), drinking/taking drugs during sex (never/one or more times), and number of sexual partners (one/two or more) in the past six months.

*PrEP adherence* was self-reported by AGYW who indicated whether they received a PrEP prescription at any time since the baseline survey. We adapted the three items by Wilson et al. [37] to address PrEP (versus antiretroviral) adherence as follows: number of days of missed doses, frequency of PrEP use, and use of PrEP as intended. We created a summary adherence score representing the past 30 days by transforming each item response into a 0- to-100-point scale and then averaging the scores. Higher scores indicated better adherence.

#### Implementation outcomes

*Reach* was measured by the proportion of AGYW-FC dyads who consented/assented to participate in the study. *Feasibility* was assessed as the proportion of AGYW-dyads in the IMARA-SA group who (a) attended at least one session, and (b) received the full 10-hour intervention. *Acceptability* was measured using brief surveys completed by AGYW and FC in the IMARA-SA group at the end of each intervention day. AGYW and FC reported their satisfaction with the intervention day, perceptions of their facilitators (e.g., whether the facilitators were knowledgeable), engagement, and learning via six items (Cronbach’s alpha = 0.58). Response options used a 5-point Likert scale (1 = not at all to 5 = extremely) for 6 six items and yes/no for 2 items. Two open-ended questions invited AGYW and FC to share what they learned and suggestions to improve the intervention. Finally, *fidelity* was measured as the IMARA-SA facilitators’ adherence to the intervention manual. Independent observers completed a checklist to denote whether each intervention activity was delivered as planned (Yes/No). Checklists were customized to each intervention day’s activities.

### Data analyses

We compared demographic and outcome variables across groups using chi-square tests for binary/categorical variables, Fisher’s exact tests where the sample sizes were small, and t-tests for continuous variables. We used chi-square tests and t-tests to examine differences between AGYW lost-to-follow-up and those who completed follow-up, as well as differences in time to follow-up between intervention groups.

We carried out an intention-to-treat analysis for effectiveness outcomes. All AGYW in both groups received HTC at follow-up, and so no statistical tests of differences were performed. We examined intervention effects on STI testing and PrEP uptake using logistic regression to obtain crude and adjusted odds ratios (ORs), 95% confidence intervals (CIs), and p values (Wald tests). For STI testing, the adjusted model controlled for age group (15–17 or 18–19 years) and STI test result at baseline. For two participants who completed STI testing at follow-up but not at baseline, we conservatively coded their baseline STI test results as positive. Treatment was confirmed for all participants who tested positive for a STI at baseline except one, and she was removed from the analysis. For PrEP uptake, we excluded AGYW who reported taking PrEP prior to baseline. The adjusted model controlled for age group.

To assess differences in sexual risk behavior outcomes by treatment group at follow-up, we used Fisher’s exact p values due to small sample sizes. We assessed intervention effects on PrEP adherence using linear regression to obtain beta coefficients, 95% CIs, and p values (Wald tests), following checks of normality and regression diagnostics (e.g., examining residuals). The adjusted model controlled for age group and baseline report of a PrEP prescription.

In all regression models, standard errors were not adjusted for clustering because AGYW did not complete all sessions with the same group. We conducted a sensitivity analysis, assuming that those who did not complete follow-up surveys or present for follow-up data collection had undesired outcomes, but the overall findings did not change. Statistical analyses were conducted using STATA 15 [38].

Implementation outcomes were summarized using percentages for IMARA-SA’s *reach* and *feasibility* and percentages and means for IMARA-SA’s *acceptability*. Open-ended responses for acceptability were thematically coded. Intervention *fidelity* ratings were averaged across observer forms to determine the proportion of activities completed as planned.

### Ethics

Written informed consent was obtained from AGYW (aged 18 and over) and separately from participating FC. In line with the South African National Health Act [39], written parental/guardian consent and AGYW assent was obtained from minors (aged 15–17 years). Where AGYW chose to participate with a female caregiver other than her parent/guardian, written consent was obtained from both the female caregiver and the parent/guardian. Ethical approval was obtained from the University of Cape Town (077/2019) and the University of Illinois at Chicago (2018–0709).

## Results

During recruitment, 147 AGYW were approached, of whom 61 (41.5%) were enrolled, completed baseline assessments, and were randomized (Fig 1). Two AGYW-FC dyads were withdrawn or removed during analyses for not meeting inclusion criteria, resulting in 59 AGYW analyzed at baseline (29 IMARA-SA, 30 control). At follow-up, survey data were analyzed for 51 AGYW (86% overall; 90% IMARA-SA, 80% control); one survey was excluded because it was collected 8 months later than the other surveys. Additionally, clinical data were analyzed for 39 AGYW (66% overall; 69% IMARA-SA, 63% control) who presented for HTC, STI testing, and/or PrEP at the on-site clinic. There were no statistical differences in the baseline characteristics of AGYW lost-to-follow-up compared to those who remained in the study (including by intervention group), or in the length of follow-up between arms.

### Baseline characteristics

Characteristics of AGYW were balanced across arms at baseline, except that a higher proportion of control versus IMARA-SA AGYW reported inconsistent condom use (p = 0.05) (Table 1). Over half of AGYW were 15–17 versus 18–19 years old (mean = 17.2 years, inter-quartile range = 16–19) and had completed some or all of secondary school (56%). One in every six AGYW reported supporting herself financially in the past year (17%). A majority (56%) were living with the participating FC, who were most often biological mothers (41%), aunts (27%), and sisters (19%).

**Table 1:** Baseline characteristics of adolescent girls and young women by treatment group.

	Total	IMARA-SA	Control	p value
Full sample	59	29	30	N/A
**Demographics**				
Age				
15-17 years old	34 (56.6%)	17 (58.6%)	17 (56.7%)	0.88
18-19 years old	25 (42.4%)	12 (41.4%)	13 (43.3%)	
Highest education achieved				
Some or all of primary school	22 (37.3%)	11 (37.9%)	11 (36.7%)	0.12
Some or all of secondary school	33 (55.9%)	18 (62.1%)	15 (50.0%)	
Higher education	4 (6.8%)	0 (0.0%)	4 (13.3%)	
Supported self financially in past year	10 (17.0%)	3 (10.3%)	7 (23.3%)	0.30
**Participation in study**				
Female caregiver participating				
Biological mother	24 (40.7%)	12 (41.4%)	12 (40.0%)	0.70
Aunt	16 (27.1%)	6 (20.7%)	10 (33.3%)	
Sister	11 (18.6%)	7 (24.1%)	4 (13.3%)	
Cousin	5 (8.5%)	3 (10.3%)	2 (6.7%)	
Grandmother	1 (1.7%)	0 (0.0%)	1 (3.3%)	
Other	2 (3.4%)	1 (3.5%)	1 (3.3%)	
**Sexual behavior**				
Ever had sex	39 (66.1%)	20 (69.0%)	19 (63.3%)	0.65
Among those who ever had sex (n=39):				
Age at first sex	15.6 (1.01)	15.6 (1.10)	15.7 (0.93)	0.57
Had sex in past 6 months	26 (66.7%)	12 (60.0%)	14 (73.7%)	0.37
Among those who had sex in past 6 months (n=27):				
Did not use a condom at last sex	11 (42.3%)	4 (33.3%)	7 (50.0%)	0.39
Used condoms inconsistently in past 6 months	14 (53.9)	4 (33.3%)	10 (71.4%)	0.05
Used substances during sex in past 6 months	18 (69.2%)	8 (66.7%)	10 (71.4%)	0.79
Had 2+ partners (versus 1 partner) in past 6 months	10 (38.5%)	5 (41.7%)	5 (35.7%)	0.76
**HTC, STI testing, and PrEP**				
HTC uptake^ (n=57)	56 (98.3%)	28 (96.6%)	28 (100%)	0.32
Tested positive for an STI^^ (n=56)	21 (37.5%)	12 (42.9%)	9 (32.1%)	0.41
Ever prescribed PrEP (self-reported)	7 (11.9%)	2 (6.9%)	5 (16.7%)	0.25
Adherence to PrEP (0=low to 100=high) (n=7)^*	70.8 (18.5)	75.0 (14.9)	69.1 (21.1)	0.74

Notes: Figures are n (%) or mean (SD). All sexual behavior variables include vaginal and/or anal sex. P values are from chi-square tests for binary/categorical variables, t-tests for continuous variables, and Fisher’s exact tests where the sample sizes were small.

^Denominator is those who were not living with HIV at baseline.

^^Denominator is those who completed STI testing (gonorrhea and/or chlamydia) at baseline.

^*Denominator is those who self-reported having ever been prescribed PrEP at baseline and were therefore asked the survey questions about adherence to PrEP.

Two-thirds of AGYW (66%) reported having ever had vaginal and/or anal sex, with an average age of 15.6 years (SD: 1.01) at first sex; 67% of these reported having vaginal and/or anal sex in the past six months. Two AGYW—one in each intervention group—reported living with HIV during baseline clinical data collection. Nearly all eligible AGYW requested HTC (98%) and received STI testing (95%) from our study team. No AGYW tested positive for HIV, but over one-third (38%) tested positive for gonorrhea and/or chlamydia. About 12% of AGYW reported that they had ever been prescribed PrEP, and these rates did not significantly differ across arms.

### Preliminary effectiveness

#### Primary outcomes

At follow-up, 39 AGYW (20 IMARA-SA, 19 control) presented for HTC, STI testing, and/or PrEP uptake (i.e., a PrEP prescription) at the clinic. Thirty-seven were eligible for HTC (not living with HIV) (20 IMARA-SA, 17 control), and all (100%) elected to be tested. STI incidence and PrEP uptake outcomes favored the IMARA-SA group but did not reach statistical significance; 34 AGYW completed STI testing at follow-up (18 IMARA-SA, 16 control), and 22% of IMARA-SA girls versus 38% of controls tested positive for an STI. Out of 45 AGYW who completed follow-up surveys, were not living with HIV, and did not report having been prescribed PrEP at baseline (25 IMARA-SA, 20 control), 68% of IMARA-SA girls took up PrEP compared to 45% of controls (Table 2).

**Table 2:** Differences in HIV testing and counseling (HTC), STI test results, and PrEP outcomes between treatment groups at follow-up.

	Sample size	Follow-up			Crude estimate	95% CI	p value	Adjusted estimate*	95% CI	p value
		Total	IMARA-SA	Control						
HTC uptake^	37									
No		0 (0%)	0 (0%)	0 (0%)						
Yes		37 (100%)	20 (100%)	17 (100%)						
Tested positive for an STI^^	34									
No		24 (70.6%)	14 (77.8%)	10 (62.5%)	1			1		
Yes		10 (29.4%)	4 (22.2%)	6 (37.5%)	0.48	(0.11, 2.14)	0.33	0.26	(0.04, 1.59)	0.15
PrEP uptake*	45									
No		19 (42.2%)	8 (32.0%)	11 (55.0%)	1			1		
Yes		26 (57.8%)	17 (68.0%)	9 (45.0%)	2.60	(0.77, 8.77)	0.12	2.83	(0.81, 9.89)	0.10
Adherence to PrEP in past 6 months**	21	58.6 (23.3)	60.7 (19.5)	54.4 (30.8)	6.26	(-16.66, 29.18)	0.57	5.00	(-17.78, 27.78)	0.65

Notes: Estimates for STI testing and PrEP uptake are odds ratios assessed via logistic regression. Estimates for adherence to PrEP are beta coefficients assessed via linear regression. Adjusted estimates include the age group (15–17 vs. 18–19 years) and the baseline value. For PrEP uptake, we adjusted only for age group (15–17 vs. 18–19 years) since we removed from the analysis those who reported having been prescribed PrEP at baseline. For PrEP adherence, we adjusted for the baseline report of having been prescribed PrEP.

^Denominator is those who came to site for HTC at follow-up and were not living with HIV (39 came to site for follow-up; 2 were removed who were living with HIV).

^^Denominator is those who completed STI testing at follow-up. Excludes one participant who completed STI testing at follow-up but did not receive STI treatment at baseline.

*Assessed as receipt of a PrEP prescription from our research team (determined by study records) or by another care provider after the baseline survey (determined by self-report). Denominator is those who completed follow-up surveys, were not living with HIV, and did not report having been prescribed PrEP at baseline.

**Denominator is those who reported having been prescribed PrEP on the follow-up survey and not on the baseline survey.

#### Secondary outcomes

Four of five secondary outcomes also favored the IMARA-SA group but were not statistically significant. Compared to controls, a smaller proportion of AGYW in the IMARA-SA group reported that they did not use a condom at last sex (44% versus 56%), used a condom inconsistently in the past six months (44% versus 56%), and used substances during sex in the past six months (50% versus 56%) (Table 3). Among those who reported receiving a PrEP prescription from baseline to follow-up (n = 21), the mean score for PrEP adherence was higher in the IMARA-SA group (60.7, SD: 19.5) compared to the control group (54.4, SD: 30.8) but this difference was not statistically significant (Table 2).

**Table 3:** Differences between treatment groups at follow-up on sexual behavior outcomes among adolescent girls and young women who report having had vaginal and/or anal sex in the past 6 months (n = 27).

	**Follow-up, n(%)**			p value*
** **	**Total**	**IMARA-SA**	**Control**	
Sample size	27	18	9	
Used a condom at last sex				
Yes	14 (51.9%)	10 (55.6%)	4 (44.4%)	0.70
No	13 (48.2%)	8 (44.4%)	5 (55.6%)	
Consistency of condom use in past 6 months	* *	* *	* *	
Always	14 (51.9%)	10 (55.6%)	4 (44.4%)	0.70
Less than always	13 (14.2%)	8 (44.4%)	5 (55.6%)	
Used substances during sex in past 6 months				
Never	13 (48.2%)	9 (50.0%)	4 (44.4%)	1.00
1+ time	14 (51.9%)	9 (50.0%)	5 (55.6%)	
Number of partners in past 6 months				
1 partner	22 (81.5%)	14 (77.8%)	8 (88.9%)	0.64
2+ partners	5 (18.5%)	4 (22.2%)	1 (11.1%)	

*Fisher’s exact test

### Implementation outcomes

#### Reach

Of those who were eligible, 100% consented or assented to be part of the study.

#### Feasibility

Intervention participation levels were high in the IMARA-SA group; 90% (n = 26) completed the first intervention day and 76% (n = 22) completed the full 10-hour intervention.

#### Acceptability

IMARA-SA evaluations were completed by 97 participants (50 AGYW, 47 FC). Eight AGYW evaluations were removed given discrepancies between the number of participants in attendance and the number who completed evaluations. This resulted in 89 evaluations analyzed, including 46 from Intervention Day 1 (85% of those who completed Day 1) and 43 from Intervention Day 2 (93% of those who completed Day 2).

Mean scores on the six Likert items ranged from 4.7 to 4.9 on a scale of 1 (not at all) to 5 (extremely). At least 76% of AGYW and FC reported being “extremely” in agreement with the acceptability statements. Nearly every participant reported understanding the material (100%) and learning something (98%). Results were consistent for AGYW and FC and therefore are presented together (Table 4).

**Table 4:** Intervention acceptability among adolescent girls and young women and female caregivers randomized to the IMARA-SA intervention, aggregating surveys across intervention days (n = 89 evaluations).

	1 (Not at all)	2	3 (Somewhat)	4	5 (Extremely)	Mean (SD)
1. How satisfied were you with today’s workshop?	0 (0%)	0 (0%)	0 (0%)	13 (15%)	76 (85%)	4.9 (0.4)
2. How knowledgeable were your group leaders about the information presented today?	0 (0%)	0 (0%)	1 (1%)	12 (13%)	76 (85%)	4.8 (0.4)
3. How comfortable were you with the group leaders who worked with you today?	0 (0%)	0 (0%)	2 (2%)	19 (21%)	68 (76%)	4.7 (0.5)
4. How much did you feel the group leaders valued what you said?	0 (0%)	0 (0%)	2 (2%)	12 (13%)	75 (84%)	4.8 (0.4)
5. How honest did you feel you could be during today’s workshop?	0 (0%)	0 (0%)	4 (4%)	15 (17%)	70 (79%)	4.7 (0.5)
6. How much did you feel you were involved or engaged in the activities? (n=49)	0 (0%)	0 (0%)	3 (3%)	18 (20%)	67 (76%)	4.7 (0.5
					**Yes**	**No**
7. Did you understand the material taught/presented in today’s workshop? (n=80)					80 (100%)	0 (0%)
8. Did you learn anything today? (n=85)					84 (98%)	1 (1%)

Score range for items 1 through 6 were on a Likert scale from 1 = not at all to 5 = extremely. All results are n (%) except for the Mean (SD) column.

In open-ended responses, AGYW described what they had learned. Almost half of responses (49%) focused on learning about communication strategies with mothers and/or partners, 17% on risky sexual behavior and condom use, 13% on PrEP, 11% on HIV/STI risk, and 10% on other topics (e.g., self-worth, coping with stress). For example:

*I learned to value my body more and to show respect to all people around me*. *I learned how to communicate in a good way with my parents and how I should respond to them when they talk*. *I learned about sexuality and how risky sex without a condom is*. *(AGYW*)

Most responses from FC (68%) focused on learnings relating to communication with their daughters, followed by learnings about HIV/STIs (13%), sexual behavior (7%), PrEP (4%), and other topics (e.g., partner violence) (8%). One FC wrote:

*I felt like today I actually woke up from not being a good enough parent to trying to be better*. *I felt closer to my daughter and also felt like a weight has been lifted off my shoulders*. *(FC*)

Both AGYW and FC expressed what the program had meant to them and their desires for it to expand its reach:

*IMARA must keep continuing to teach young women about their selves*. *(AGYW*)

*They should go outside Philipi and give other areas these kinds of sessions*. *(FC*)

Additional representative quotes are in S1 Table.

#### Fidelity

In the IMARA-SA group, observation was conducted in 8 out of 12 sessions hosted with daughters (67%) and 6 out of 12 sessions hosted with mothers (50%). The proportion of activities completed as planned revealed a treatment fidelity of 94.8%.

## Discussion

This pilot study responds to the critical need to identify effective approaches to reduce HIV and STI risk among AGYW in sub-Saharan Africa [40]. At follow-up, STI incidence and PrEP uptake, along with four of five secondary outcomes, favored IMARA-SA versus control participants but did not reach statistical significance. These results suggest that the IMARA-SA intervention has important potential to reduce STI incidence, improve PrEP uptake and adherence, and mitigate sexual risk behavior among AGYW. Moreover, HTC uptake was 100% in both arms at follow-up, suggesting that offering HTC through the intervention could help fill a gap in the availability of acceptable services for AGYW in this setting. These favorable findings point to the potential value of engaging the female caregivers of AGYW in efforts to promote positive sexual health decision-making. They also support ongoing efforts to expand PrEP use and adherence, increasingly recognized as a critical strategy to prevent HIV acquisition among South African AGYW [41, 42].

We found promising evidence for reach, feasibility, acceptability, and fidelity. Consistent with Glasgow et al. [22], reach was evident in that 100% of eligible AGYW-FC dyads agreed to participate. Newer formulations of the RE-AIM framework underscore the importance of participant representativeness when evaluating an intervention’s impact [22]. This study focused on one of the most vulnerable populations in South Africa for HIV and STI acquisition—AGYW—thereby amplifying the significance of these preliminary findings.

IMARA-SA was also feasible to implement and highly acceptable to participants as indicated by strong attendance and positive ratings and responses to open-ended questions among both AGYW and FC. Of note, feasibility and acceptability are “leading indicators” of intervention success [43] and key predictors of adoption [44]. The intervention was further delivered with high fidelity, and AGYW and FC reported favorable views of their facilitators. Fidelity is critical to attributing observed effects to the intervention [45] and positive perceptions of facilitators are linked with better program outcomes [46]. Facilitators in this study came from the same community as participants and did not have prior specialized training or education. This points to the potential scalability of IMARA-SA if found to be effective in an RCT.

Given the small sample size of this pilot study, we used Fisher’s Exact Tests to assess sexual risk behavior outcomes, and we used logistic regression to evaluate effects on STI testing and PrEP uptake. These results should be interpreted cautiously and underscore the importance of conducting a fully powered trial. The small sample size may account for the lack of statistically significant differences between groups on primary and secondary effectiveness outcomes, particularly given the consistent results favoring IMARA-SA across seven of the eight outcomes. Likewise, the strength of the control group’s health promotion program may have resulted in underestimates of intervention effects compared to what would have been observed with a “standard of care” control group. Participant retention at follow-up was high for surveys (86%), but only moderate for clinical data (66%). COVID-related lockdowns and restrictions hampered our ability to conduct in-person visits. The research site/team has instituted numerous procedures to prevent similar disruptions in data collection in the future (i.e., staff vaccinations, mandatory masking). Furthermore, the PrEP and sexual risk behavior outcomes relied largely on retrospective self-report, which are subject to social desirability and accurate memory [47]. Still, evidence suggests a relatively high correlation between self-reported sexual behavior and STI incidence [48].

## Conclusions

Findings from this pilot study demonstrate promising potential for the IMARA-SA intervention to reduce HIV/STI risk among AGYW in South Africa. Results suggest that a family-based HIV/STI prevention program spanning 10 hours over two days is feasible to deliver, able to reach a highly vulnerable population, acceptable to AGYW and FC alike, and can be implemented with fidelity by community members with adequate training and supervision. The favorable findings for IMARA-SA support efforts currently underway to conduct a fully powered RCT within this setting.

## Supporting information

S1 Checklist CONSORT 2010 checklist of information to include when reporting a randomised trial*. (DOC)

S1 Fig Curriculum summary. Summary of curriculum content for the IMARA-SA intervention group versus the health promotion control group.
(TIF)

S1 Table Experiences with the IMARA-SA program. Sample open-ended responses from adolescent girls and young women and female caregivers.
(DOCX)

S1 Data Provides the data analyzed for this study. (XLSX)

S1 Protocol Trial protocol. (PDF)
