# Supplementary figures and images for: Preliminary effectiveness and implementation outcomes of the IMARA-South Africa sexual health intervention on adolescent girls and young women: A pilot randomized trial
Source: PLOS Glob Public Health. 2023 Feb 15;3(2):e0001092. doi: 10.1371/journal.pgph.0001092 (PMC10022073; doi:10.1371/journal.pgph.0001092)

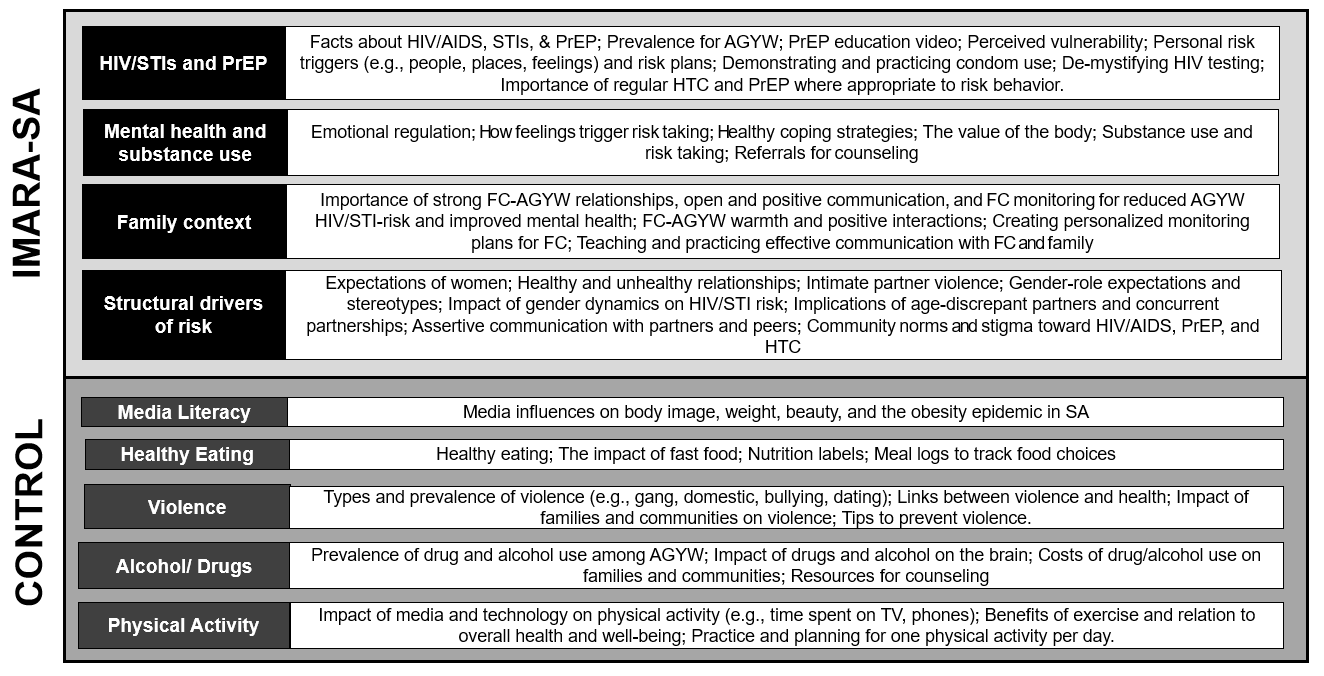

Supplement: S1 Fig — Summary of curriculum content for the IMARA-SA intervention group versus the health promotion control group. (TIF) [file pgph.0001092.s002.tif]
