# Supplementary material for: Preliminary effectiveness and implementation outcomes of the IMARA-South Africa sexual health intervention on adolescent girls and young women: A pilot randomized trial
Source: PLOS Glob Public Health. 2023 Feb 15;3(2):e0001092. doi: 10.1371/journal.pgph.0001092 (PMC10022073; doi:10.1371/journal.pgph.0001092)
Supplement: S1 Table — Sample open-ended responses from adolescent girls and young women and female caregivers. (DOCX) [file pgph.0001092.s003.docx]

**Preliminary effectiveness and implementation outcomes of the IMARA-South Africa sexual health intervention on adolescent girls and young women**

**S1 Table. Experiences with the IMARA-SA program.** Sample open-ended responses from adolescent girls and young women (AGYW) and female caregivers (FC).

| **Learnings from IMARA-SA** |
| --- |
| “[I learned] how to communicate with my mother. From now on, I know now to talk with my mother about sex.” (AGYW)  “I learned more about self-value and setting goals. Also the different communication techniques and I learned why it is important to use a condom every time you have sexual intercourse.” (AGYW)  “I learned about how to use a condom and how to communicate with your boyfriend. I learned how to use PrEP and how to face your challenges without alcohol.” (AGYW)  “I learned the difference between aggressive and passive communication. I also learnt that being aggressive doesn’t solve anything. For you deal with a situation, you need to be calm and be sure about what you say.” (AGYW)  “[I learned] to engage in a good manner with my daughter and be honest and calm. To know when is the right time to talk.” (FC)  “[I learned] how to build a relationship between me and my daughter. From now on, I am prepared to talk about anything with my daughter.” (FC)  “[I learned] that when communicating, listen to the other person. Remember to be calm when approaching certain situations. Be open to your kids….value other people’s point of view.” (FC) |
| **Support for IMARA-SA** |
| “I liked everything. The facilitators were so open and explained everything perfectly.” (AGYW)  “IMARA is helping us. It must carry on doing this.” (AGYW)  “For me, I would prefer [IMARA] stays the same because they bring so much joy.” (AGYW) |
| **Desires for the future of IMARA-SA** |
| “What I think IMARA should do…[is] go to schools and talk to school kids and tell them about this program. Because most of the children out there do not have bonds with their mothers, so it will help them a lot.” (AGYW)  “It should be done all around the world because it is really about a very important issue.” (AGYW)  “To go to other places and countries to teach people about IMARA because other people need to learn about this. It is very important. It teaches a lot.” (FC) |
