# Supplementary material for: Preliminary effectiveness and implementation outcomes of the IMARA-South Africa sexual health intervention on adolescent girls and young women: A pilot randomized trial
Source: PLOS Glob Public Health. 2023 Feb 15;3(2):e0001092. doi: 10.1371/journal.pgph.0001092 (PMC10022073; doi:10.1371/journal.pgph.0001092)
Supplement: S1 Protocol — (PDF) [file pgph.0001092.s005.pdf]

UNIVERSITY OF CAPE TOWN FHS015: SECTION C RESEARCH PROTOCOL

# Multilevel Comprehensive HIV Prevention Package for South African Adolescent Girls and Young Women

**Short Title: IMARA**

**Version 5.0**

**14 May 2020**

**Funding Agency**

**National Institute of Child Health and Human Development**
**Grant Number: 1 UG3 HD096875-01**

**Key Collaborating Organizations**
**Center for Dissemination and Implementation Science and Healthy Youths Program**
**University of Illinois at Chicago**
**USA**

**Desmond Tutu HIV Foundation, Cape Town, South Africa**

KEY ROLES AND CONTACT DETAILS ........ 3
*Investigators Signature Page* ........ *6*
**PROTOCOL SUMMARY ........ 7**
**1. INTRODUCTION ........ 9**
1.1 BACKGROUND AND RATIONALE ........ 9
**2. STUDY DESIGN ........ 15**
2.1 STUDY OVERVIEW, STUDY DESIGN, AREA AND SETTING ........ 15
2.2 STUDY PARTICIPANTS AND PROCEDURES FOR PHASE 1 ........ 16
*Curriculum Adaptation* ........ *17*
*PHASE 1: Curriculum adaptation procedures* ........ *18*
2.2.1 ELIGIBILITY, RECRUITMENT AND INFORMED CONSENT/ASSENT PROCEDURES FOR AIM 118
*Eligibility and inclusion criteria* ........ *18*
*Recruitment and Enrolment* ........ *19*
PARTICIPANT ENROLMENT IN AIM 1 ........ 19
*Retention Procedures.* ........ *22*
*Additional Tracking Procedures.* ........ *23*
A) AIM 2: PILOT TESTING WITH 50 AGYW-FC DYADS (100 TOTAL PARTICIPANTS) TO ESTABLISH ACCEPTABILITY AND FEASIBILITY IN PREPARATION FOR THE RCT IN PHASE 2 ........ 20
*Recruitment, enrolment and procedures for AIM 2* ........ *20*
PROCEDURES FOR PREP ........ 21
*Formulation* ........ *21*
*Storage* ........ *21*
*PrEP initiation and dosage* ........ *22*
*ART initiation* ........ *22*
*Community PrEP referral clinics.* ........ *22*
DATA COLLECTION PROCEDURES ........ 23
BIOLOGICAL SPECIMEN COLLECTION, HANDLING AND TESTING ........ 25
DATA REDUCTION, DATA ANALYSIS AND EXPECTED OUTCOMES ........ 26
*Expected outcomes* ........ *26*
*Data Reduction and Analyses.* ........ *26*
PARTICIPANT REIMBURSEMENT ........ 26
**3. HUMAN SUBJECTS PROTECTION ........ 26**
3.1: INFORMED CONSENT ........ 27
3.2. RISK/BENEFITS TO PARTICIPANTS ........ 28
3.3 PRIVACY, DATA, CONFIDENTIALITY: ........ 32
3.4. DATA PROTECTIONS ........ 33
3.5. PROCEDURES TO MONITOR ADVERSE EVENTS ........ 33
3.6 POLICY ON DATA SHARING ........ 34
3.7 PUBLICATION POLICY ........ 34
3.6 STUDY TIMELINES ........ 34
**1. REFERENCES ........ 35**

## *Key Roles and Contact Details*

| Site Project Principal Investigator |
|---|

| | |
|---|---|
| Linda-Gail Bekker, MD, PhD<br>Desmond Tutu HIV Centre<br>University of Cape Town<br>Werner and Biet Building North, Anzio Road,<br>Mowbray, Cape Town, 7925 | Tel: +27 21 650 6959<br>Mobile: +27 83 266 2876<br>Fax: +27 21 650 6963<br>E-mail:linda-gail.bekker@hiv-research.org.za |
| **International Principal Investigator** | |
| Geri R. Donenberg, PhD<br>Centre for Dissemination and Implementation Science<br>Department of Medicine<br>University of Illinois Chicago<br>818 S. Wolcott Ave, SRH- 3rd Floor<br>Chicago, IL 60613<br>United States | Tel: +1 312-355-1857<br>Fax:+1 312-996-6893<br>E-mail: gerid@uic.edu |
| **Co- Investigators** | |
| Millicent Atujuna, PhD<br>Study sub-investigator<br>Desmond Tutu HIV Foundation<br>University of Cape Town<br>Werner and Biet Building North, Anzio Road,<br>Mowbray, Cape Town 7925 | Tel: +27 21 650 6961<br>Mobile: +27 825187551<br>Fax: +27 21 650 6963<br>E-mail:Millicent.atujuna@hiv-research.org.za |
| Surrey Walton<br>Pharmacy Systems, Outcomes and Policy<br>University of Illinois Chicago<br>1603 W. Taylor Street, 845 SPHPI, Chicago, IL 60612<br>United States | |
| Erin Emerson<br>Centre for Dissemination and Implementation Science<br>Department of Medicine<br>University of Illinois Chicago<br>818 S. Wolcott Ave, SRH- 3rd Floor, Chicago, IL 60613<br>United States | |
| Lauren Fynn, MA<br>Desmond Tutu HIV Foundation<br>Masiphumelele research offices<br>Guinea Fowl Rd, Sunnydale, Fish Hoek,<br>Cape Town, 7975, South Africa | Tel: +27 21 785 3121<br>lauren.fynn@hiv-Research.org.za |
| Natasha Crooks, PhD, RN<br>Department of Women, Children, and Family Health Science | |

| | |
|---|---|
| University of Illinois at Chicago<br>845 S. Damen Ave., MC 802,<br>Chicago, IL, 60612<br>United States | |
| **Project coordinator and budget personnel** | |
| Kate Merrill, PhD<br>IMARA Project Director<br>Centre for Dissemination and Implementation Science<br>University of Illinois at Chicago<br>Department of Medicine<br>818 S. Wolcott Ave, SRH- 3rd FloorChicago, IL 60613 | |
| Marisela Ramirez<br>IMARA Budget Personnel<br>Center for Dissemination and Implementation Science<br>Community Outreach Intervention Projects<br>University of Illinois at Chicago<br>Department of Medicine<br>818 S. Wolcott Ave, SRH- 3rd Floor<br>Chicago, IL 60613 | |
| Emily Pela<br>IMARA Budget personnel<br>Centre for Dissemination and Implementation Science<br>Community Outreach Intervention Projects<br>University of Illinois at Chicago<br>Department of Medicine<br>818 S. Wolcott Ave, SRH- 3rd Floor<br>Chicago, IL 60613 | |
| Sheily Busisiwe Ndwayana<br>Desmond Tutu HIV Foundation<br>University of Cape Town, Werner and Biet Building North, Anzio Road, Mowbray, Cape Town 7925 | Tel: +27 21 650 6960<br>Sheily.ndwayana@hiv-research.org.za |

**Investigators Signature Page**
**Version 5.0**
**Desmond Tutu HIV Foundation**

The signature below constitutes the approval of this protocol and the attachments, and provides the necessary assurances that this *study* will be conducted according to all stipulations of the protocol, including all statements regarding confidentiality, and according to all applicable legal and regulatory requirements and regulations as well as ICH and SA GCP guidelines.

I, as the Principal Investigator, agree to conduct this study in full accordance with the provisions of this protocol. Publication of the results of this study will be governed by DTHF policies.

I have read and understand the information in this protocol and will ensure that all associates, colleagues, and employees assisting in the conduct of the study are informed about the obligations incurred by their contribution to the study.

Principal Investigator:

| | |
|---|---|
| ……………………………………………… Signature | Name: Professor Linda-Gail Bekker |
| | Date: 3 June 2020 |
| | |

## PROTOCOL SUMMARY

| | |
|---|---|
| **Purpose:** | The main objectives of the proposed study are to compare the effectiveness and cost-effectiveness of IMARA adapted for the SA context (IMARA-SA) vs. a mother-daughter general health promotion program matched for time and attention and adapted to the SA context (FUEL-SA). This study is funded under the UG3 UH3 program, where by specific UG3 milestones have to be met in order to transition to UH3 aims. This application will focus on UG3 aims only. |
| **Primary objectives** | **<u>UG3 Aims Transitional milestones (24 months study)</u>**<br>• Systematically adapt IMARA for South African 15-19 year old adolescent girls and young women (AGYW) and their Female Caregivers (FC). This will be based upon completed theatre group testing with 48 AGYW-FC dyads.<br>• To conduct pilot testing with approximately 50 AGYW-FC dyads with follow up at 6- and 12-months, in order to establish processes and feasibility for phase II RCT. Key milestones for this phase of the study include obtaining participant STI incidence > 20% (with 100% receiving DOT), uptake of HTC > 70%, uptake of PrEP prescription > 50% and retention at 6 months of > 80%.<br><br>**<u>UH3 Aims (36 months)</u>**<br>• Conduct a methodologically-rigorous 2-arm RCT comparing the impact of FUEL-SA and IMARA-SA on 525 15-19 year-old SA-AGYW and their FC on STI and HIV incidence, HTC and PrEP uptake, sexual risk behaviours, and important theoretical mediators. Compared to FUEL-SA, we expect SA-AGYW in IMARA-SA to report increased condom use, have fewer incident STI and HIV infections, be more likely to adopt PrEP and HTC, and demonstrate greater improvement on theoretical mediators of risk at 6- and 12-month follow up.<br><br>• Evaluate the costs and cost-effectiveness of IMARA-SA compared to FUEL-SA with respect to the acquisition of STI, and where possible, HIV considering power limitations.<br><br>• Explore participant views and beliefs about PrEP and HTC for SA-AGYW. We will conduct 40 key informant interviews with SA-AGYW and FC about PrEP and explore facilitators and barriers. |
| **Design** | The study uses mixed methods which aim to address the study objectives, with qualitative and more participatory methods during the adaptation of the study curriculum, and understanding participant views and beliefs about PrEP. The study also utilizes RCT methods to evaluate family based scalable interventions to prevent HIV and STIs among AGYW aged 15-19 years old. |
| **Study site** | Recruitment will occur in Klipfontein/Mitchells Plain (K/MP) health subdistrict and neighbouring areas including crossroads, Philippi, Gugulethu, and from our Tutu |

| | |
|---|---|
| | teen mobile unit. Participants will be invited to come to the Desmond Tutu HIV Foundation satellite research site in Philippi village for consent procedures, medical study components and group sessions. |
| **Study drugs** | Oral Pre-exposure prophylaxis (Truvada) (one month supply); STI treatment: N. gonorrhea – Ceftriaxone 250 mg injection (Rocephin®) AND Azithromycin (Zithromax™) 1g orally; C. trachomatis – Azithromycin (Zithromax™) 1g orally; T. vaginalis – Metronidazole (Flagyl™) 2g orally; HIV testing and counseling (HTC) |
| **Study population** | 15 – 19-year-olds, AGYW and their Female Caregivers (24 years and older) |
| **Sample size** | A total of ~1250 participants are expected to be enrolled across all study components as follows:<br>1. Up to 96 participants in aim 1 theatre testing component<br>2. Up to 100 participants in the pilot study<br>3. Up to 1050 participants in the RCT<br>4. Up to 40 participants in the qualitative component |
| **Treatment regimen** | Referral to ART clinics for HIV positive participants; Referral for PrEP for HIV negative participants with a one month supply provided at enrolment if participants are willing to take PrEP. |
| **Study duration** | 5 years (2 years UG3 feasibility) and (3 years UH3 RCT) |
| **Duration of enrolment and accrual** | 36 months |
| **Primary endpoints** | An adapted and tailored SA IMARA curriculum;<br>Impact of IMARA on HIV and STI incidences;<br>PrEP uptake;<br>HTC uptake;<br>Cost effectiveness and scalability of IMARA;<br>Sexual risk behaviour (condom use, number of partners) |
| **Key secondary endpoints** | AGYW-FC relationship (warmth, acceptance, monitoring, permissiveness);<br>AGYW-FC communication (quality, frequency);<br>HIV knowledge (HIV/STI transmission, prevention);<br>HIV-related Stigma;<br>HIV and STI attitudes;<br>AGYW mental health (depression, anxiety, trauma);<br>AGYW and FC knowledge, attitudes and beliefs about PrEP and HTC;<br>Adherence (PrEP and ART);<br>Gender roles and gender-based violence;<br>Drug use;<br>Partner communication; and<br>Power relationship dynamics. |

# 1. INTRODUCTION

## *1.1 Background and Rationale*

**Reducing new HIV infections among adolescent girls and young women (AGYW) is a global public health priority.[1]** Progress in the AIDS epidemic has been uneven, and gross inequities persist.[2] SA-AGYW have one of the highest incident rates globally,[3] acquiring HIV at twice the rate of their male peers, and seroconvert on average 5 – 7 years earlier than males their age.[4] Similarly, the prevalence and incidence of sexually transmitted infections (STI) which facilitate HIV acquisition[5, 6] are higher among AGYW than males their age.[7-9] Among SA-AGYW, 15-19 year-old females account for over two thirds of new HIV infections,[10] but only 15% know their HIV status.[11] Sexual behavior is the primary mode of HIV transmission in SA,[4, 12, 13] but up to 60% of SA-AGYW report not using condoms at first sex and 17% report never using a condom.[14, 15] New infections continue to outpace access to and availability of antiretroviral treatment, and thus, primary prevention remains the most viable approach to stem new transmissions.[16] Yet, achieving an AIDS-free generation depends on addressing the needs of SA-AGYW.[1, 2]

**SA's HIV epidemic reflects a unique and complex tapestry of the country's political, economic, historical, and sociocultural landscape.[17-20]** The history of apartheid, urbanization, unemployment, poverty, and forced migration have shaped the health of SA.[17] The country's growing rates of mental illness, escalating drug and alcohol use, HIV/AIDS stigma, past AIDS denialism, misinformation about HIV, and previous governmental failures to address these problems have fuelled SA's epidemic. Reducing risky sexual behavior among young SA is a high priority, but countrywide efforts have been largely ineffective at reducing new transmissions among AGYW.[21-25] Most of SA's youth-targeted HIV prevention programs focus almost exclusively on individual-level behavior change,[26-28] but AGYW's persistent HIV disparities are explained by a multiplicity of social and structural inequities that both shape and constrain HIV-risk behaviors and continue to drive incident infections.[29-34] Multi-level combination HIV prevention packages that are integrated, synergistic, and tailored to the local epidemiology and cultural context are more likely to achieve and sustain maximum reductions in HIV-risk and vulnerability.[35-42] This application addresses these gaps by evaluating a multi-level comprehensive HIV prevention package that includes components advocated by PEPFAR and the WHO (condom programming, behavioral strategies, HTC, sexual and reproductive health, mental health, HIV/AIDS stigma, community empowerment, PrEP, and gender-based violence) and leverages the mother-daughter relationship and communication to promote positive sexual and reproductive health in 15-19 year-old SA-AGYW.

The topic under investigation necessitates inclusion of children, in this case with a focus on adolescents aged 15-19 years. This is specifically critical for prevention science particularly in South Africa where the mean age at sexual debut is 16 years and, therefore, we want to ensure that these adolescents, some of who are entering this age receive the necessary interventions. Moreover, this target age group is among those most vulnerable to acquiring HIV and STIs. Research indicates that adolescent girls and young women (AGYW) are experiencing new infections more than males their age. Developmentally, this is also a critical age where adolescents are mastering abstract thoughts, are negotiating independent decision making, and

are beginning to engage in risky sexual behaviour. Targeting this group is useful for prevention science as we are able to leverage interventions that speak directly to this developmental stage including family interventions that are particularly important to mitigating risk in this group. Moreover, because this a prevention intervention, targeting this group before they engage in high-risk sexual behaviours or may have recently initiated sexual activity allows us to examine the efficacy of the intervention with young girls. Without these adolescent participants, we will be unable to accomplish the study objectives to develop and test efficacious HIV risk and STI prevention interventions that use a family-based approach. For this reason, the inclusion of adolescent girls and young women is necessary in the proposed research study.

**This proposal is guided by an ecological framework**[32] **that emphasizes the intersection of individual, sociocultural, and structural determinants of SA-AGYW's sexual health and behavior (see Figure 1).** The framework addresses key drivers of HIV among SA-AGYW with implications for HIV prevention. **Individual-level factors are personal characteristics** that influence individual behavior, including knowledge, attitudes, beliefs, skills, and mental health. HIV knowledge and awareness have increased in SA youth,[28] but young people believe they are at low risk of infection[15, 43-45] and widespread misconceptions remain.[15, 45-47] Simbayi et al.[46] found that 29% of SA youth believed washing their genitals after sex reduces HIV-risk, and 39% said AIDS is caused by spiritual forces. Among SA 9th graders, 14% reported that men who have sex with a virgin can cure themselves of HIV, and an additional 40% were unsure.[48] Beliefs and attitudes toward PrEP and HTC (e.g., people will think I am gay) continue to influence low uptake and adherence. Mental health problems are related to increased HIV-risk, poor sexual decision making, difficulty coping with distress, and diminished HIV prevention effects.[49-53] SA surveillance data show moderate rates of lifetime anxiety (16%), mood (10%), or substance-related (13%) disorders among adults,[54] but mental health disorders peak during adolescence[55, 56] making this developmental period a critical intervention point. Few HIV prevention efforts with SA youth address mental health.[22, 57] Individual factors driving HIV-risk must be integrated into comprehensive approaches to achieve sustained shifts in the epidemic.[22, 58-60]

**Figure 1: Individual, sociocultural, and structural drivers of HIV risk and prevention for SA AGYW**

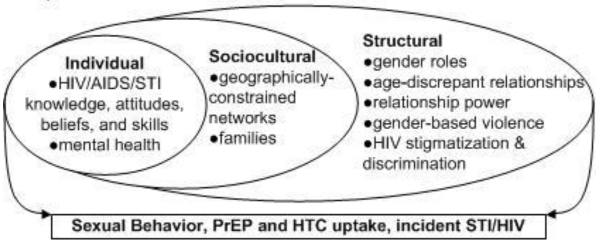


**Sociocultural-level factors are proximal environmental characteristics that impact HIV-risk and vulnerability**. Geographically constrained sexual networks, common in SA black and mixed race communities, exacerbates HIV-risk by restricting sexual partner choices within one's community.[61, 62] Where high rates of STI and HIV exist in one's sexual network, transmission is more likely.[63, 64] In SA, concurrent sexual partnerships are common, tolerated, and even encouraged for males, providing opportunities for rapid spread of the virus.[65] These contextual realities are vital to address in HIV prevention programming for at least two reasons. First, understanding the importance of social and community level risks may de-

stigmatize individual AGYW behavior. Second, the information can empower AGYW to make sexual decisions within their context of risk.

Families provide the first and most important socialization experience for sexual and reproductive **health.** Primary female caregivers (referred to as “mothers” in this application) play a central role in AGYW’s sexual health and development. Studies in the US indicate that maternal warmth and attachment, close family relationships, parental involvement and support, and positive parenting predict more consistent condom use, less exposure to HIV-risk situations, and later sexual debut among adolescents.[51, 66-69] Mother-daughter communication that is open, receptive, and comfortable is associated with reduced sexual experience and less risky sexual behavior in the US[51, 70-76] and in Africa.[77-82] Families are vital support systems that can be effective partners in reducing SA-AGYW risk and promote uptake of effective HIV prevention strategies, including HTC, linkage to HIV care, PrEP, and safer sex in a de-stigmatized way. As a powerful center of family life, the SA matriarch may serve as a critical catalyst for change. Capitalizing on the mother-daughter relationship, strengthening mother-daughter communication about sexual behavior, and improving young women’s healthy sexual decision making represents a structural intervention that can alter the current HIV-trajectory for SA-AGYW. SA is especially suited to family-based HIV prevention interventions that strengthen family structures and empower parents.[83] The investigative team (Donenberg, PI) recently completed a small pilot study of Project STYLE[84] adapted for SA with 80 13-18 year-old males and females and their parents in psychiatric care in a black township in the Cape metropolitan area. Following the two-day, group-based workshops with 8 – 10 parent-teen dyads each, parents routinely reported appreciating the opportunity to learn how to communicate more effectively with their youth around sexual topics and to learn from other parents in the group. To date, however, families are a relatively untapped resource in HIV prevention efforts,[85, 86] especially with AGYW, and therefore offer significant promise as an opportunity to strengthen the HIV prevention toolbox.

**Structural-level factors are “deeply embedded manifestations of historical inequities and injustices”**[32] including relationship power differentials, gender-based violence,[13, 26, 87] intergenerational sex, hierarchical gender roles, HIV/AIDS stigma, and economic vulnerability.[13, 88] Gender dynamics drive SA-AGYW HIV-risk and failure to use effective prevention strategies.[89-96] Traditional *African gender roles* that depict women as men’s property and subordinates[15] and expect monogamy for women and concurrency for males,[97-100] are entrenched cultural values that make it difficult for SA-AGYW to insist on safer sex.[13, 101, 102] Many men are involved in overlapping sexual partnerships,[103] a practice implicated in HIV transmission.[104, 105] Over 40% of SA young people reported that they knew or suspected that their partners were having unprotected sex with other people,[14] and 10-30% reported having sex with more than one partner at a time.[15, 103] *Age-discrepant relationships*[106, 107] often driven by economic inequality and sociocultural norms are implicated in acquiring HIV and STI.[101] SA girls’ first and ongoing sexual partner is typically an older male.[108] SA-AGYW with older partners were four times more likely to test positive for an STI,[9] report transactional sex,[109] and have high HIV infection rates.[110] Older male partners place AGYW at a major disadvantage conferring less *relationship power,*[111, 112] minimal access to HIV education, and less ability to reject advances.[107] AGYW are also less likely to use or negotiate condoms with

older partners,[89, 111, 113, 114] because asking to use condoms can challenge the balance of relationship power, imply infidelity and lack of trust, challenge traditional female roles, and jeopardize the emotional support and financial stability offered by older partners. *Gender–based violence (GBV)* among SA-AGYW is higher than every other region in sub-Saharan Africa,[115 14, 97] and is related to early sexual debut,[116] high rates of STI,[9] and greater HIV risk.[117-119] In SA, 12% of new HIV infections were attributable to GBV in a single year.[120] Unfortunately, the perceived benefits of age discrepant relationships often outweigh perceived risks, as older male partners confer status, emotional support, and financial security.[90, 101, 104, 121-127] HIV-related stigma and discrimination are powerful deterrents to HIV prevention and care,[128, 129] including reduced likelihood of HTC among SA youth,[130] decreased uptake and adherence to PrEP,[131, 132] and poor retention in HIV treatment.[133] Discussions of gender dynamics and integrating stigma-reduction strategies into program designs are imperative to address HIV and STI transmission in the SA context.

**Comprehensive HIV prevention strategies that address individual, sociocultural, and structural drivers of HIV among SA-AGYW are essential to shift the tide of the epidemic.** Most programs for SA youth (e.g., Beyond the Awareness,[134] loveLife, Soul City[135]) focus almost exclusively on individual-level behavior change[26-28] and suffer significant methodological weaknesses.[21-25, 136-138] Most of these interventions are school-based[85, 139] and show stronger effects for 10-25 year-old males than females.[138] There has been relatively scant attention to young people's social context, including gender-power dynamics[1] or family factors.[58] loveLife, one of the largest and most expensive prevention campaigns ever implemented in sub-Saharan Africa, led to increased awareness and perceived vulnerability but did not reduce viral spread.[140] The Mpondombili Project for 14-17 year olds in rural Kwazulu-Natal emphasized gender-role norms, inequalities, and social norms about sexuality,[141] but outcomes on youth behavior have not been published. HealthWise South Africa, delivered to 8th and 9th graders, revealed increased perceptions of condom availability but no effect on sexual behavior.[142] Kirby et al.[143] and Visser[144] reported increased awareness and knowledge of HIV, but weak to moderate effects on adolescent sexual risk taking. Finally, evidence for the government sponsored Life Skills program has been mixed,[143, 145, 146] and prior studies involving family members[147-149] have not examined adolescent behavior change. In an extensive review of school-based programs, Mukoma and Flisher[25] concluded that effects on adolescent behavior change have not been realized.

**Family-based programs hold exceptional promise to improve uptake and sustainability of HIV prevention strategies,** including PrEP and HTC, by addressing the sociocultural and structural drivers of HIV and challenging gender dynamics and HIV stigma in a safe and culturally appropriate venue.[149-152 153, 154] Mothers can continue to deliver prevention messages after the formal intervention ends and tailor messages to AGYW's developmental stage. Programs that improve the quality of mother-daughter conversations about sexual decision making, teach parents to use accurate information, and challenge cultural taboos that are barriers to mother-daughter communication about sex[86] can increase AGYW's prevention activities. Only 20% of SA youth receive information about sex from their parents, and almost 50% of SA girls reported that they could not openly discuss sexuality with their parents.[14] Yet, girls said their parents were the best source of information about sex and were receptive to parent-teen discussions.[155] African mothers reported needing and wanting

accurate knowledge to feel confident discussing sex with their children.[156] Youth report conversations with parents as ill-timed, and emphasizing fear of STI, the dangers of sex, and premarital sex as a sign of "waywardness".[156-158] African parents sometimes deliberately mislead children by using inexact terms, e.g., "women's thing" for vagina or "irresponsible behavior" for sexual activity.[158] Group-delivered family-based interventions (as proposed in this study) can build on the power of collective and community support, change social and cultural norms to favor HIV prevention and uptake of established (HTC) and new (PrEP) technologies, and create a sense of unity and collective efficacy to stem AGYW HIV and STI infections.[32, 159]

**HTC and PrEP are essential components of comprehensive HIV prevention.** Research that optimizes uptake and adherence to PrEP and HTC in SA-AGYW[160] is critical to promote equitable access to all HIV scientific advances. HTC is widely recognized as an important tool to prevent HIV transmissions, but only 15% of 15-19 year-old AGYW in Southern Africa have been tested and received their results.[11] Innovative strategies are needed to encourage HTC.[161] The Desmond Tutu HIV Foundation (DTHF) (the site for the proposed project) is at the forefront of efforts to engage adolescents in HIV prevention through adolescent friendly testing services where youth naturally gather (e.g., youth centers, drop-in sites).[161] Linking adolescent-friendly HTC with family-based prevention can strengthen efforts to facilitate uptake leveraging parental support. Likewise, PrEP is highly effective in reducing incident infections[162-166] and can prevent HIV in young women[167] by equipping them with greater control and agency over their sexual lives.[168] The DTHF and Co-PI Bekker have been leading some of the most important PrEP studies in SA. However, PrEP efficacy depends on near perfect adherence for women[162, 163, 169-174] to achieve the protective concentration in the female genital tract.[175] Disappointing results from clinical trials with women[172, 173] have been traced to poor adherence, with younger, unmarried, and the highest risk women least likely to adhere.[162, 164, 176] While PrEP appears to be acceptable to older women,[168, 172, 176, 177 87, 173, 178-185] it is unclear if PrEP is acceptable or feasible for AGYW, as they have not been included in research due to regulatory and parental permission issues.[1, 186] By including mothers in this study with SA-AGYW, we avert these barriers, thereby allowing us to explore AGYW views and beliefs about HTC and PrEP and leveraging the mother-daughter relationship to address the advantages of HTC, PrEP uptake and adherence.

**The next step in the science of HIV prevention for SA-AGYW is rigorous evaluation of combination prevention strategies,[42] but the cost of implementing programs cannot be ignored**. Cost containment is at the forefront of HIV prevention efforts in Africa where resources are increasingly limited. Establishing the cost-effectiveness of combination HIV prevention packages is essential if they are to be sustained over time.[187, 188] Systematic reviews suggest that HIV prevention is highly cost-effective[189, 190] with the greatest impact and cost-effectiveness for populations with HIV incidence > 3 per 100 person years.[189] In 2012, HIV incidence for SA-AGYW was 2.54%.[4] Although PrEP has the potential to avert a significant number of infections[136] and be cost-effective for adults, [191, 192] no empirical studies to date have evaluated the cost-effectiveness of comprehensive HIV prevention for 15-19 year-old SA-AGYW that includes a family-based intervention. The proposed study includes a cost analysis on STI infections, and thus, will offer important information to decision-makers who must decide how best to allocate economic resources to maximize the impact of funded

programs. Although power to detect effects on HIV is limited, where possible, we will explore the cost-effectiveness on incident HIV infections.

**This study will test the effectiveness and cost-effectiveness of a multi-level HIV-prevention package for SA-AGYW that addresses individual, sociocultural, and structural drivers of HIV-risk.** We will adapt a highly promising innovative family-based HIV prevention program – *Informed Motivated and Responsible about AIDS* (IMARA) -- that showed a 54% reduction in incident STI infections among 14-18 year-old African American girls at one-year follow up compared to a matched control group (i.e., FUEL) (see Table 1). IMARA is based on three programs listed in the Centers for Disease Control Compendium of *HIV Prevention Programs with Demonstrated Evidence of Effectiveness,*[193] SISTA[194-198] and SiHLE[199, 200] for black women and Project STYLE for families in mental health care.[199] During the adaptation stage of the proposed study, we anticipate adding content to address HIV-related stigma, PrEP information and education, HTC and PrEP uptake and adherence within the SA context, and strengthening content regarding the risks of partner concurrency within geographically constrained areas and GBV.

**Table 1. Effect of IMARA on STI incidence at 12 months**

| | N | Baseline n (%) | 12-months n (%) | RR (95% CI) | p-value |
|---|---|---|---|---|---|
| IMARA | 53 | 10 (18.9) | 8 (15.1) | 0.46 (0.22-0.9 | .049 |
| FUEL | 46 | 9 (19.6) | 15 (32.6) | | |

**The proposed study has high potential impact and a strong scientific premise.** SA-AGYW have been left behind in the strides made in the AIDS epidemic. The unrelenting disparity of HIV among SA 15-19 year-old females[154] requires an expansion of prevention options that address individual, sociocultural, and structural drivers within the SA context.[136] Ample evidence indicates that focusing on vulnerable populations and high-incidence locations will realize the greatest gains in altering epidemiological trajectories.[201] While changing behavior can reduce HIV and STI acquisition,[30] research on evidence-based interventions with strong evaluation designs and cost-effectiveness data is woefully lacking for SA-AGYW, especially those capitalizing on the strengths and assets of families. This study targets SA-AGYW, an exceptionally high-risk population in a country with the highest number of incident HIV infections in the world. To date, no comprehensive, multi-level, family-based HIV prevention program exists for SA-AGYW that targets individual, sociocultural and structural drivers of risk to decrease HIV and STI incident infections and increase HTC and PrEP uptake. The long-term significance of the study is to reduce HIV and STI transmissions among SA-AGYW.

**The proposed study is innovative in a number of important ways**. First, the proposed HIV prevention package goes beyond individual behavior change and addresses the broader sociocultural (implications of geographic isolation, family relationships) and structural (gender dynamics, HIV-related stigma) drivers of HIV-risk and vulnerability among SA-AGYW, a population at exceptional risk for incident HIV and STI infections. Second, the multi-level intervention involves mothers, an untapped yet potentially valuable resource in HIV prevention for SA-AGYW. By leveraging the mother-daughter relationship, we are adding a tool to the HIV prevention toolkit with exceptional promise at reducing risk and increasing uptake of prevention strategies, including HTC and PrEP. Third, rigorous tests of HIV prevention packages for SA-AGYW are largely absent, and the proposed, well-designed study will carefully evaluate intervention effectiveness and cost-effectiveness allowing for methodological rigor and reproducibility. Fourth, ample evidence suggests that the effects of

prevention efforts with young people decay over time. This study has the potential to sustain positive outcomes for SA-AGYW as mothers continue to deliver prevention messages (including support for routine HTC and PrEP adherence) after the formal intervention ends and tailor them to AGYW's developmental stage. Fifth, this study will include biomedical interventions (PrEP and HTC) in addition to behavioral and social approaches. Little is known about the feasibility or acceptability of PrEP or HTC among 15-19 year-old SA females given parental permission regulations. By engaging mothers in this project, we will be able to explore AGYW views and beliefs about PrEP and HTC. Sixth, IMARA directly addresses the impact of mental health on HIV-risk, teaching emotion regulation strategies that emphasize thoughtful rather than impulsive responses to distress. This study provides an opportunity to mitigate the growing rates of mental health problems in SA. Finally, few economic evaluations of comprehensive HIV prevention packages are available for SA-AGYW. To our knowledge, this will be the first study to carefully evaluate the STI acquisition cost-effectiveness of a family-based HIV prevention program for SA-AGYW to inform and guide the most efficient use of limited resources.

Thus, the overall objectives of the study are to **compare the effectiveness and cost-effectiveness of IMARA adapted for the SA context (IMARA-SA) vs. a general health promotion program matched for time and attention and adapted to the SA context (FUEL-SA)**. We will:

1) **Systematically adapt and pilot test IMARA and FUEL for 15 – 19 year-old SA-AGYW and their FC**.

2) **Conduct a methodologically-rigorous 2-arm RCT comparing the impact of FUEL-SA and IMARA-SA on 525 15-19 year-old SA-AGYW and FC on STI and HIV incidence, HTC and PrEP uptake, sexual risk behaviors, and important theoretical mediators**. Compared to FUEL-SA, we expect SA-AGYW and FC in IMARA-SA to report increased condom use, have fewer incident STI and HIV infections, be more likely to uptake PrEP and HTC, and demonstrate greater improvement on theoretical mediators of risk at 6- and 12-month follow up.

3) **Evaluate the costs and cost-effectiveness of IMARA-SA** compared to FUEL-SA with respect to the acquisition of STI, and where possible, HIV considering power limitations.

4) **Explore participant views and beliefs about PrEP and HTC for SA-AGYW**. We will conduct 40 key informant interviews with SA-AGYW and FC about PrEP and explore facilitators and barriers.

# 2. STUDY DESIGN

## *2.1 Study overview, study design, area and setting*

The complete study will be conducted in two phases (**Table 2**). Phase 1 will adapt and evaluate feasibility of the IMARA program in preparation for a Randomized Control Trial (RCT) in phase 2. We request ethics approval for phase 1 of the study. A separate application for phase 2 will be submitted once phase 1 is complete. Therefore, the methods presented below focus on phase 1 of the study.

**Table.2**

| Phase 1 study methods (UG3) | Phase 2 study methods (UH3) (*separate Ethics application)* |
|---|---|
| A mixed methodology<br>1. Theatre testing (group discussions) and adapting the curriculum for SA youth n=48 (AGYW-FC dyad)<br>2. Pilot testing with approximately 50 AGYW-FC dyad to establish feasibility for the RCT in phase 2 | A mixed methodology<br>1. RCT with AGYW-FC dyad n=525<br>2. Evaluate cost effectiveness<br>3. Interviews with (AGYW-FC dyad) n=40 |

**Study area and Setting.**

We will implement the study in the Klipfontein/ Mitchells Plain (K/MP) health sub-district of the Western Cape metropolitan area and neighbouring communities including Gugulethu, Crossroads and Philippi. Generally, the selected communities a high HIV/TB burdened, low resource, and high-density community. Recent data on 15-19 year-old females in the sub-district revealed high rates of incident STI (7.4%), STI prevalence (52%), intergenerational sex (20%), concurrent partners (27%) and low rates of condom use (42%).[202] Thus, this is an ideal setting for this study. We will also recruit participants from our Tutu Teen mobile unit (TTMU) which targets adolescents.

***2.2 Study Participants and procedures for phase 1***

Phase 1 aims to ***(a)*** systematically adapt IMARA and FUEL for 15 – 19 year-old SA-AGYW and their FC. We will enrol 48 AGYW-FC dyads (96 total participants) and conduct theatre testing to guide curriculum adaptations for the SA context. ***(b)*** We will conduct pilot testing with approximately 50 AGYW-FC dyads (~100 total participants) to establish feasibility in preparation for a 2-arm RCT in phase 2. The theatre participants will not participate in the pilot study precisely because these participants will have been involved in curriculum adaptation sessions, have the advantage of the material already. Critical, however, is that the pilot study participants are randomly assigned to receive the intervention or to the control arm and it will be difficult to isolate those theatre testing participants who already know the intervention curriculum.

### Interventions, intervention curriculum and adaptation

Our goal in aim 1 is to adapt and pilot test the revised IMARA curriculum for the South African context. IMARA (see Table 4) was derived from three CDC demonstrated evidence-based interventions -- SISTA (Sisters Informing Sisters about Topics on AIDS),[194] SiHLE (Sistering, Informing, Healing, Living, and Empowering),[195] and Project STYLE (Strengthening the Youth Life Experience) and adapt it for the South African context.[84] [203] Separate FC and AGYW groups run simultaneously, cover parallel content, and address individual, sociocultural, and structural drivers of HIV-risk and vulnerability from a gendered and ethnic/cultural perspective. There are also joint FC-AGYW activities designed to enhance mothers' credibility as a resource for HIV/STI prevention, and facilitate the practice of new communication skills. Additional activities address conflict negotiation, assertive communication, and strengthening the AGYW-FC relationship. The material is presented in a group format to promote structural change by building community norms for prevention and reducing HIV stigma. Each session begins with an icebreaker and/or poem to enhance ethnic and gender pride. IMARA's goals and motto are presented to emphasize strong FC-AGYW relationships, foster sisterhood and community empowerment, build group cohesion, and increase motivation for HIV prevention. Each FC and AGYW signs the IMARA pact to confirm her commitment to the program. IMARA is delivered over two days separated by one week to allow time to assimilate information. At the end of day 1, FC and AGYW receive homework to complete during the week. Woven throughout IMARA is the impact of mental health problems and alcohol and drug use on HIV-risk (e.g., condom use while drunk). Preliminary data for IMARA revealed a 54% reduction in STI incidence at 12-month follow-up compared to the FUEL control group (see Table 1).

**Table 4. IMARA curriculum**

| Theoretical Constructs | IMARA Activities |
|---|---|
| **Individual Factors** | |
| *Ethnic and gender pride* | Poems: A Room Full of Sisters, Phenomenal Woman; Music, movies, TV images that portray black women (positive, negative); Successful black women: Name that woman! Young, black & female; Role models; Black women who shaped history |
| *Knowledge, attitudes, beliefs*<br>· HIV/AIDS, HTC, PrEP | HIV/AIDS jeopardy; Virus carrier handshake; High, moderate, low risk situations; Videos – HIV facts; Identifying risk triggers (people, places, feelings); Developing individual risk plans; LIPSTICK (acronym for condom use steps); HTC information and role play |
| *Mental health and emotion regulation* | Feelings as triggers for risk behavior; Feeling thermometer; Healthy coping; Links between mental health and HIV-risk behavior; What matters most – distinguishing values; Taking care of you; Value of my body; Poem: Still I Rise; Phenomenal Woman scavenger hunt |
| **Sociocultural Factors** | |
| *Family context*<br>· Parental monitoring<br>· Mother-daughter attachment and communication | Personalized parental monitoring plans; Passive, aggressive, and assertive communication w/role plays; Observed mother-daughter conflict discussion w/feedback; Rephrase it-game – Using I-statements; Get to know you game; Reverse role-plays; Successful black women game; Mother-daughter LIPSTICK; Public service announcements; Mother-daughter values discussion; Mother challenge – scenario; Understanding normal adolescent development |
| **Structural Factors** | |
| *Gender dynamics*<br>• Gender roles, beliefs, GBV<br>*Partner relationships*<br>• Partner concurrency<br>• Partner communication<br>*HIV/AIDS Stigma* | Pieces and parts of relationships; Gender-role stereotypes; Recognizing healthy vs. unhealthy relationships; Concurrent partnerships in the context of HIV/AIDS; Choosing healthy relationships; What is gender-based violence? Partner selection/types & implications for HIV-risk; Dating older males – risks and advantages; KISS – Keep It Simple Sister; Comebacks to pressure lines; Passive, aggressive, and assertive communication w/role-plays; Changing community norms toward HIV/AIDS and HTC. |

In the initial adaptation process, we will invite our Community Advisory Board (CAB) members will review the IMARA curriculum and offer suggestions for revisions and tailoring to the South African context. These suggestions will be discussed by the investigative team, and where appropriate, incorporated into a revised curriculum. The CAB members will also recommend modules and components of the curriculum to "theatre test" or try out with AGYW and FC for feedback.

Trained individuals who speak both English and Xhosa, the language most spoken in the area and by our CAB members, will deliver the IMARA curriculum that will be theatre tested with

participants. These group leaders will employ a variety of interactive and experiential activities to deliver the identified components of IMARA to AGYW and FC. First, they will present a component/activity, and then they will stop to facilitate a discussion with AGYW and FC and obtain recommendations for change. They will continue to follow this process until all components/activities of the curriculum are presented and feedback obtained. Following the theatre testing, the investigators will discuss the recommendations and make decisions on content modification, session timing and appropriate and suitable days for sessions.

**Health Promotion Control Group.**

The control program is matched for time and is similarly delivered to AGYW and FC. The control program promotes healthy living by encouraging good nutrition, exercise, and informed consumer behaviour. The control group does not explicitly address HIV/STI prevention, but we will present basic information about HIV/AIDS and other STI given the high risk for the AGYW. We will also undertake a similar process of adaptation to ensure that the control program is relevant for the SA context, working closely with CAB only. For example, we will invite suggestions of other SA-relevant non-HIV-related health issues that might be added to the program. Investigators will meet to discuss recommended revisions to the control program, make decisions about content and make changes to the curriculum as appropriate.

**PHASE 1: Curriculum adaptation procedures**

- Present IMARA's curriculum topics and activities the DTHF Community Advisory Board (CAB), and request feedback regarding cultural relevance and acceptability.
- Investigative team will review feedback from the CAB and decide what to revise prior to theater testing.
- Theater testing will occur with 48 AGYW-FC dyads.
- Investigators will discuss a detailed summary of the theater testing for overall themes, content suggestions, and general recommendations; They will discuss feedback about each activity.
- Study coordinator will develop recommendations to tailor specific activities based on feedback from AGYW and FC during theater testing.
- Re-convene the DTHF CAB to review the detailed summary of the theater testing along with recommendations for tailoring the IMARA curriculum derived from the investigator meeting; Invite additional feedback.
- Invite input from "Topical Experts" -- individuals who bring unique expertise as needed.
- Integrate final changes and translate amended materials, including consent and assent documents.
- Conduct a pilot test with approximately 50 AGYW-FC dyads.

### *2.2.1 Eligibility, Recruitment and Informed Consent/Assent procedures for aim 1*

**Eligibility and inclusion criteria**

AGYW will be: a) black or mixed race; b) 15-19 years-old; c) residing in K/MP; and d) speak isiXhosa and English or a combination of these languages as these are the primary languages of the area. Female caregivers will be: a) identified by AGYW as a FC; b) 24 years and older; c) living with or in daily contact with the AGYW; and d) speak similar languages. FC and AGYW

must agree to participate as a dyad, but AGYW refusal will supersede FC consent. AGYW will be excluded from the study if they: a) are unable to understand the consent/assent process; b) do not speak English, isiXhosa, because instruments are normed for these languages; and/or c) do not have a female caregiver. If there is more than one eligible AGYW in a family, we will ask FC to select one to maintain independent observations. Consent/assent forms will explicitly state the exceptions to confidentiality and these will be verbally reviewed with FC and AGYW. Where an AGYW reports abuse or neglect or suicidal ideation or attempts, a clinician at the DTHF site will be consulted and appropriate referrals and reporting made as per DTHF standard operating procedures (see Human Subjects).

**Recruitment and Enrolment**

For Aim 1 (theatre testing) we will recruit 48 AGYW-FC dyads (96 total participants) to participate in discussion groups. For aim 2 (pilot testing) we will recruit approximately 50 AGYW-FC dyads (~100 total). AGYW and FC will be recruited from community events, public libraries, mobile services (teen truck; tutu tester) and by going door to door in Philippi, Crossroads and Gugulethu communities. Our initial recruitment strategy will involve sending out flyers in English and Xhosa describing the study. Flyers will also be posted at our current community based sites and at our mobile services. Given that our sample size for phase 1 is relatively small, our strategy will involve:

**(1)** purposively approaching a female parent/caregiver (FC) who cares for a 15-19 year old girl, explaining the study and inviting participation. If the FC is willing to participate with a AGYW, we will collect her contact details and information regarding availability to schedule a visit to our site for consent and study enrolment. We will also collect details for the AGYW for further contact. Should FC and AGYW be available for recruitment at the same time, we will explain the study to both of them and then separately ask for their willingness to participate. The nature of the study requires that both FC and AGYW are comfortable to participate in the study together. In cases where the AGYW prefers someone other than her mother to participate in the study, such as an aunt or older sister, we will seek the mother's permission to enrol the AGYW with an alternative FC. Likewise, in cases where mother prefers the AGYW to participate with someone else, preference will be given to the AGYW to choose a FC with whom to participate. In both cases, we will obtain (1) consent from the parent/caregiver allowing her AGYW to participate with another FC for her choice (24 years and older), (2) consent from the participating FC, and (3) assent for AGYW < 18 years old and consent for AGYW $\geq$ 18 years old.

**(2)** Approaching AGYW, briefly explaining the study, and determining eligibility. If AGYW are interested, we will only at this point obtain contact details of the parent/caregiver. We will contact the parent/FC to describe the study and gauge interest. We will schedule the dyad for consent, assent and enrolment into the program. Information about random assignment will be explained once during the consent process.

***Participant enrolment in aim 1***

Once 6-8 FC-AGYW dyads are scheduled and arrive at the site, we will:

- Complete consent and assent forms separately with AGYW and FC.

- Convene the group. Describe and demonstrate the specially selected IMARA activity, stop and request feedback about its relevance and acceptability for SA, then invite recommendations for adaptation.
- Sessions will be audio recorded and transcribed.
- The study coordinator will summarize the comments in writing (checking notes against the tapes) for review and discussion by the investigators and DTHF CAB.
- Formal qualitative analyses of the audiotapes are not required given the nature of the feedback.

a) ***Aim 2: Pilot testing with ~50 AGYW-FC dyads (~100 total participants) to establish acceptability and feasibility in preparation for the RCT in phase 2***
**Recruitment, enrolment and procedures for AIM 2**

We will pilot test the revised IMARA curriculum with ~50 new AGYW-FC dyads, using the same eligibility and recruitment procedures described above. Groups will include approximately 6-8 dyads and participants will be followed for 12 months. The procedures for aim 2 are described next:

- We will schedule 8-10 dyads in each cohort to comprise one group. Upon arrival at the research site (Philippi village), trained staff will review consent and assent documents, ensuring comprehension and evaluating if participants are willing to participate.
- Following the consent/assent process, all AGYW and FC will complete a baseline assessment via computer-assisted self-interview (approx. 1 ½ hours) and indicate their desire to test for HIV and STIs and willingness to take PrEP. If willing to test for HIV and STIS, both AGYW and their FC will receive HIV/STI counselling and testing. AGYW and FC have the option to refuse testing and still remain in the study.
- Randomization (control vs. intervention): We will randomize participants to treatment arm upon completion of the baseline assessment so as not to lose families due to bias in advance. Approximately 25 dyads will be randomly assigned to the health promotion group and ~25 dyads to IMARA. Randomization will follow the fish-bowl method, where equal numbers of RED and WHITE cards will be placed in the fish bowl. AGYW will select a card. The two intervention groups will take place in different rooms. The control and IMARA curricula are matched for time and attention.
- The first group session (approx. 3 hours) will be delivered immediately following randomization. Participants will be at site for 6.5 hours for their first session activities, including the baseline assessment and intervention.
- Participants will return one week later for day 2 of the intervention.
- The total time of the intervention is approximately 12 hours.
- Participants who agreed to test for HIV will be given their results at the end of day 1. If HIV positive, referral for care in the regular public ART clinics will be provided. For those who agreed to test for STIs, results will be ready by session 2. If positive, the DTHF nurse will provide STI risk reduction counselling and treatment. We will request the partner be treated as well.
- For participants who are HIV negative and opt to take PrEP in our baseline survey, we will provide a one month PrEP prescription with a referral to public, youth friendly clinics currently offering PrEP. We will draw blood for safety monitoring of creatinine in

accordance with the national guidelines. We will additionally test blood draws for Hepatitis B, in line with national guidelines, among participants who opt to take PrEP at the 6- and 12-month follow-up points. Urine will be tested for pregnancy. Renal function (e.g., creatinine clearance, CrCl) will be tested and all results will be supervised by a medical member of our team. Participants will receive the results with referral documents for the next dose of PrEP.

- At each follow up data collection point and after the second session, we will re-invite individuals for HIV and STI testing. We will also re-invite participants to receive PrEP, unless they accepted PrEP from us at a previous data collection timepoint, were linked to care, and are receiving PrEP from a clinic. Should they be willing to test and to use PrEP, the same procedures as above will apply. At the end of their first month post PrEP initiation and referral, the team will follow up on PrEP and ART uptake at clinics including where they plan to seek PrEP or ART services. We will also ask about whether side effects participants are experiencing when they come for their second visit a week after the first session. We encourage all our participants to contact us should they experience any side effects once they have started using PrEP. During follow-up visits at 6- and 12- months, we will additionally use dried blood spots (DBS) to assess adherence to PrEP among those who have accepted PrEP.
- We will implement extensive retention and tracking procedures (see retention and tracking procedures section below).
- We will re-interview AGYW and FC 6- and 12-months after baseline for preliminary evidence of feasibility and acceptability, particularly with regard to the procedures for HTC, STI and PrEP uptake, and linkage to care for treatment.
- We will collect written and verbal feedback from participants at the end of each session. Investigators will meet and discuss final proposed changes which will be incorporated into study procedures and the curriculum.
- We will prepare for the RCT and submit an application to Ethics for phase 2 of the study.

The transition from phase 1 to phase 2 of the study is based on meeting specific milestones achieved in the both aim 1 and aim 2 of phase 1.

### *Procedures for PrEP*

#### Formulation

Young women who are willing to use PrEP in the pilot will be immediately provided a one month supply of a **TENVIR-EM** fixed dose combination tablet containing 200mg emtricitabine and 300mg tenofovir disoproxil fumarate (TDF). **TENVIR-EM** is manufactured by CiplaMed. **TENVIR-EM** has been approved by the US Food and Drug Administration (FDA) for treatment of HIV-1 infection and PrEP to reduce the risk of sexually acquired HIV-1 in adults.

#### Storage

TENVIR-EM will be stored as per WHO good pharmacy practice (GPP) and good distribution practice (GDP). The tablets will be stored in their original containers on shelves in secure rooms in FPD offices in the study communities. The rooms will only be accessible to authorized study staff. The temperature in the rooms will be controlled by air conditioning between 15$^{O}$C

and 30$^{O}$C. Room temperature will be monitored twice daily and readings will be documented on a temperature control log. Expiry dates of medication will be monitored monthly. Any problems encountered with stored medication will be reported to the study pharmacist (Siraaj Adams) who will provide guidance on appropriate resolution steps.

**PrEP initiation, dosage and adherence**

PrEP initiation will be done by the Project Medical Officer /Clinical Nurse Practitioner. These staff members have worked on previous PrEP studies and understand the standard operating procedures for PrEP. We will procure our one month PrEP prescription from Iyeza health (ltd) and we will work directly with the Pharmacist at Iyeza health. On the day of PrEP initiation a medication dispensing record will be signed by study staff and the participant. Participants will be encouraged to take TENVIR-EM once daily at a specified time with or without food. If a participant forgets to take TENVIR-EM at the specified time, it may be taken later in the day, however only one tablet can be taken within a twelve-hour period. Participants will be able to voluntarily discontinue PrEP at any time during follow up. Study staff will furnish participants with an information leaflet detailing the correct usage of the medication, appropriate contact information in case side effects are experienced, and practical tips to aid adherence. At the month 6 and 12 follow up visits, those who accepted to use PrEP will be asked to provide dried blood spot (DBS) samples to test drug levels as a measure of PrEP use and adherence.

**ART Treatment and initiation**

For ART, participants will be referred to public health clinics nearest to their residence for further tests and ART initiation.

**Community PrEP referral clinics.**

Currently, PrEP is offered to a cohort of sexually active, HIV-negative female adolescents, aged 15 to 24 years, through six selected primary health care services with adolescent youth friendly services (AYFS) in the Klipfontein and Mitchells Plain Health Subdistricts, Cape Town, South Africa. These include crossroads 1, crossroads 2, Inzame Zabantu, Gugulethu, Phumlani and Zamonthle clinics and the Tutu Teen Truck. Our staff members are already involved in this process and will able to monitor participants referred to these clinics for PrEP.

**Retention Procedures for Aim 2.**

We will use the same meticulous tracking procedures that we have employed in past studies to reduce attrition and sample bias.[204-206] We will conduct extensive phone/text/WhatsApp, or in-person contacts to address scheduling issues and barriers and remind participants of their assessment and session appointments. We will contact AGYW and mothers the day before sessions and assessments to confirm attendance. At baseline, we will confirm AGYW's birthdate, email, phone numbers, and home address, as well as the phone numbers, email, and home addresses of at least 3 people who will always know where they are and could help us locate them in the future. We will telephone/ text/ WhatsApp AGYW and mothers monthly to update our contact information. We will give thank you cards following each assessment to demonstrate our appreciation and personalized birthday cards with a small gift (i.e., magnet),

as prior research suggests that gifts sustain interest in the study and increase motivation to stay in touch with us.[207, 208]

**Additional Tracking Procedures.**

If we do not reach AGYW or FC after 1 week and at least 3 attempts, we will follow several steps used successfully in our previous research 1) If the phone number is disconnected, we will contact people from participants' records; (2) If AGYW were recruited through a site (vs. outreach), we will contact the health facility for assistance; (3) We will send research assistants to the family's last known address.[208] (4) If the AGYW no longer lives at the address, we will ask their contacts to help us locate them.

***Data Collection Procedures***

In addition to theatre testing and curriculum adaptation data collected verbally and through written notes, we will collect baseline data for the pilot using Qualtrics software on tablet computers. Participants will read the surveys on their own with the assistance of our trained data collection staff who are present to conduct the actual survey with participants if they are unable to read, or with the assistance of Audio Computer-Assisted Self Interview (ACASI). . A paper-based survey will be in hand and ready for use should we experience any challenges with the electronic versions. AGYW and FC (N=100 total) will be tested for three STI (chlamydia, gonorrhoea, trichomonas). A medical officer will arrange single dose antibacterial treatment for all participants who test positive for an STI. We will also offer HTC and HIV results to each participant. We will offer PrEP at the baseline assessment, and AGYW and FC who test negative for HIV and/or express a desire to take PrEP will be referred to a medical officer, who will facilitate care at the site.

The 6- and 12-month surveys may be administered either in person or via telephone. Participants may be asked only a subset of interview questions, depending on time and availability. Participants will also be asked a set of questions that were not included on the baseline survey about the COVID-19 virus. For surveys administered in person, data collection and clinical assessments will follow the same procedures as detailed above. For surveys administered via telephone, after obtaining consent/assent, study staff will ask participants each question and record their responses using Qualtrics software on tablet computers. Participants will be informed that they will be given the opportunity to complete HTC, STI, and/or PrEP clinical assessments when they are next on site.

**Baseline survey Questionnaire (see Appendix B).** Most of the measures have been used extensively with SA adolescents and caregivers and demonstrate acceptable psychometric properties in isiXhosa.

a) **Participant Demographics**. For AGYW < 18 years old, we will ask caregivers to provide residential and e-mail addresses, home and cell phone numbers, and contact information for at least 3 people who can help us locate families in the future. We will also collect maternal age and education, family income, AGYW's age, and family structure. We will collect this information directly from 18 and 19 year-old AGYW.

**B) Predictors and Mediators.** Individual-Level. (1) HIV/AIDS/STI Knowledge, Attitudes, Beliefs, and Skills. AGYW and mothers will complete the *HIV/AIDS Knowledge Survey*, a measure of transmission routes and prevention strategies[209] and AGYW will complete the *HIV/AIDS and STI Attitudes and Beliefs Survey* measuring: (a) general attitudes; (b) norms regarding prevention; (c) prevention intentions; (d) attitudes toward preventive acts; and (e) prevention skills (see Mukoma et. al., 2009).[210] (2) Mental Health and Emotion Regulation. Caregivers will complete the *Child Behavior Checklist (CBCL)* and AGYW will complete the *Youth Self-Report (YSR < 18 years) or Adult Self-Report (ASR ≥18 years)*. All three are widely-used and validated measures in isiXhosa of young people's mental health problems and generate raw and T-scores for internalizing (e.g., sadness, anxiety) and externalizing (e.g., fighting, swearing) syndromes.[211-213] AGYW will complete the *UCLA PTSD Index* to assess exposure to trauma and violence,[214] and they will complete the 6-item *Structured Interview for Disorders of Extreme Stress* to report emotion regulation.[215] (3) PrEP and HTC knowledge, Attitudes, and Adherence. AGYW will indicate their knowledge and willingness to take PrEP using items from Hoff et al.,[216] and they will report on their attitudes toward HTC using Kalichman and Simbayi's[217] 5-item scale (both used and validated in South Africa). We will inquire about linkage to care (yes/no) for PrEP and HIV, and where appropriate we will assess adherence to PrEP and ART using the 3-item scale by Wilson et al.[218] Mothers will complete similar items about their attitudes toward HTC and PrEP for AGYW. Sociocultural-Level. Family context. AGYW will report on the strength of their attachment to mothers using the *Inventory of Parent and Peer Attachment*.[219, 220] AGYW and mothers will indicate the level of parental monitoring, supervision and permissiveness using the *Parenting Style Questionnaire*[221] and on the quality and quantity of their HIV-risk related communication.[222 71, 223] Structural-Level. (1) Gender dynamics. AGYW will report on their partners' age, perceptions of control over condom use, partners' resistance to condom use, and financial dependence on partners.[90] Items from the *Sexual Relationship Power Scale (SRPS)* will measure perception of relationship control and decision-making dominance.[224] The *Power and Attitudes in Relationships* (PAIR) scale assesses gender roles and norms, perceived need to be in a relationship, women and men's division of household responsibility, and sexual assertion and power in decision-making.[225] We will measure partner concurrency with items validated by Kalichman, Ntseane, Nthomang et al.[226] and Harrison, Cleland, & Frohlich[227] in SA. Gender-based violence will be assessed using items from the *WHO Multi-Country Study on Women's Health and Life Experiences.*[228] We will examine partner sexual communication using items adapted from the *Sexual Risk Behavior Questionnaire*[229] and Miller et al.[222] Items ask about a list of topics (e.g., using condoms), how often they were discussed, and whether the conversation was open and comfortable. (2) HIV/AIDS stigma. AGYW and mothers will complete the 9-item AIDS-Related Stigma Scale validated in isiXhosa and English. The scale is internally consistent and time stable.[230] Participant Satisfaction. At the end of both workshop days, AGYW and mothers will complete an evaluation of the program.

**Survey questions added for 6 and 12-month surveys (see Appendix 1)*:*** We will ask a set of questions about the COVID-19 virus at 6 and 12 months. These measures will assess the extent to which the outbreak has impacted participants' lives and behaviors, including their sexual behavior, alcohol and drug use, and access to clinical care. Questions will also explore what participants have done to cope with COVID-19. Questionnaire items were adapted from the COVID-19 Exposure and Family Impact Survey,[231] the COVID-19 Questionnaire-Child Self-Report Primary version from the Environmental Influences on Child Health Outcomes (ECHO),[232] the CoRonaviruS Health Impact Survey (CRISIS),[233] and the iTech/ATN survey.

***Biological Specimen collection, handling and testing***

*Biological specimen collection*. We have carefully considered availability of procedures for age-appropriate biologically verified HIV and STIs. We have sought out the latest available but least invasive methods based on preparatory research of available test kits, assays, lap space, and pricing. As such, we have chosen finger prick HIV tests and urine tests for STIs (chlamydia, gonorrhea, trichomonas). We will use trained and certified counsellors, clinic and lab teams to gather HIV/STI biological data and to conduct referrals for confirmatory testing and treatment. For participants interested in PrEP, our staff are also trained to draw blood, conduct creatinine assessments, and test for Hepatitis B and DBS samples for PrEP adherence. These specimens will be used to monitor STI incidence > 20% (with 100% receiving DOT), uptake of HTC > 70%, uptake of PrEP prescription > 50% and retention at 6 months of > 80%. While HIV tests will be done at site, urine and blood specimens will be sent to BARC for testing. We will offer AGYW PrEP at each assessment, unless the participant has accepted PrEP from us during a previous data collection timepoint, been linked to care, and is receiving PrEP from a clinic.

STI. At each assessment, we will collect urine to screen for *N. gonorrhoeae*, *C. trachomatis*, and *T. vaginalis*. We will give participants collection containers and escort them to a private, secure room in which to produce the specimen. Staff will decant the urine to centrifuge tubes labelled with unique, anonymous, subject-sample identifiers and store them in refrigerators. The BARC laboratory will use nucleic acid amplification technologies (NAAT) for STI testing[234, 235] and email the results using a password protected server to us within one week. AGYW who test positive for an STI will be offered free single-session directly observed therapy by our Clinical nurse practitioner (CNP) and a medical officer on site with the following antimicrobials: *N. gonorrhea* – Ceftriaxone 250 mg injection (Rocephin®); *C. trachomatis* – Azithromycin (Zithromax™); All HTC procedures will comply with the DTHF standard operating procedures.

Our staff and counsellors at our site are extensively trained and highly experienced conducting HTC with youth and adults. At baseline, HTC will be conducted following the workshop so as not to interfere with the day's activities. At follow-up, HTC will occur at the end of the assessments so as not to interfere with other survey materials. Urine samples will be sent to the BARC laboratory for testing and an HIV rapid test will be conducted. AGYW who test positive for HIV will be immediately linked to care at ART public clinics and an appointment for follow-up care will be arranged. The project coordinator working closely with the CNP nurse will track appointment attendance by reaching out to our referral clinics. An SOP is already in place where PrEP uptake data is obtained from these clinics and we will work with the existing

SOP to obtain our participants PrEP uptake information. For STI and HIV results, we will maintain subject-sample ID linkages in confidential logs kept in secure, locked storage. The DTHF has been using these procedures for many years with no adverse events. We will offer Pre-Exposure Prophylaxis (PrEP) to AGYW and FC at each assessment point. For interested participants, we will schedule an appointment at the DTHF clinic and assist with transportation or attendance, as needed, to the initial appointment

### *Data reduction, data analysis and expected outcomes*

#### Expected outcomes

AGYW will report on their risk behavior and provide biological specimens for STI and HIV testing. **(1)** HIV/STI Risk Behaviors. *AIDS-Risk Behavior Assessment (ARBA)*[236] is a computer-assisted structured interview of self-reported sexual behavior, drug use, and HIV status derived from 5 well-established measures,[237-242] and used extensively in published research. The ARBA employs a skip structure so that questions initially answered "no" are not followed by more detailed questions. We will use specific indicators (e.g., number of partners), frequency measures, count variables (e.g., number of unprotected sex acts), and composite scores to assess risky sex. We will also calculate lifetime sexual behavior and the ratio of protected to unprotected sex acts. The measure has been used with SA adolescents. **(2)** PrEP uptake. **(3)** HIV and STI incidence.

#### Data Reduction and Analyses.

We will generate frequencies and summary statistics of predictor and outcome variables to screen and clean the data. We will check the success of randomization by comparing intervention groups on baseline variables using chi-square tests for categorical variables and t-tests or nonparametric Wilcoxon rank-sum tests for continuous variables. In subsequent analyses, we will control for predictor variables that are not balanced across arms at baseline. We also will check for differences between AGYW in IMARA-SA and FUEL-SA in subsequent analyses. We will create summary scores for AGYW on the following variables: (1) sexual activity, HIV and STI incidence, PrEP and HTC uptake; (2) individual drivers (mental health/emotion regulation; HIV/AIDS knowledge, attitudes, beliefs, skills; PrEP and HTC attitudes and beliefs); (3) sociocultural drivers (partner concurrency; family context); and (4) structural drivers (gender dynamics; HIV/AIDS stigma). We will conduct analyses on individual outcome indicators and on composite scores comprised of multiple indicators for the pilot study in aim 2.

### *Participant reimbursement*

Participants will not be paid to participate in the intervention, but they will be reimbursed for their time and transport: AGYW and FC will each receive R120 for the theater test and R130 for the pilot test at each study visit.

## 3. HUMAN SUBJECTS PROTECTION

All human subject procedures conform to the recommendations Procedures follow the "Ethics in Health Research: Principles, Processes and Structures – 2015" guidelines, as mandated

by the South African National Health Research Ethics Council. This application proposes to conduct phase 1 of the study which will adapt the intervention curriculum and pilot test all procedures that will be conducted in phase 2 RCT. During phase 1, we will first conduct theater testing with 48 AGYW/FC dyads and then pilot test the feasibility of the intervention with approximately 50 additional dyads. Inclusion and exclusion criteria. AGYW will be: a) black or mixed race; b) 15-19 years-old; c) residing in K/MP; and d) speak isiXhosa, English or a combination as these are the primary regional languages. AGYW may or may not be sexually active and information about their sexual activity will not be shared with caregivers, reducing consent/assent barriers. FC will be: a) selected by the AGYW and agreed upon by the mother/caregiver; b) 24 years and older; c) living with or in daily contact with the AGYW; and d) speak similar languages. FC and AGYW must agree to participate as a dyad, but AGYW refusal will supersede caregiver consent. We will include HIV-infected and uninfected FC and AGYW. AGYW and FC will be excluded if they: a) are unable to understand the consent/assent process; and b) do not speak English, isiXhosa because instruments are normed for these languages. AGYW will be excluded if they do not have a FC. Participants in phase 1 (theater testing, pilot test) will be excluded from phase 2. If there is more than 1 eligible AGYW in a family, we will randomly select 1 to maintain independent observations. If AGYW or FC become pregnant during the course of the study, they may remain enrolled in the study. We will link participants who become pregnant over the course of the study to prenatal care. Our trained staff will provide these services or refer participants to appropriate clinics.

### *3.1: Informed Consent*

Informed consent will be obtained from all participants. Participants will be asked to provide written consent to enrol in the theatre testing. We enrol new participants in the pilot study, necessitating a different consent form that requests consent for biological specimens to test for STI and if desired HIV. We will state in the consent documents how long the samples will be stored and what these will be used for. The informed consent and assent forms will include information about the study and individual requests for consent/assent of the participants to participate in the study. Forms will emphasise that participation is voluntary and participants are free to withdraw from the study at any stage without any disadvantage to them. The information on the consent/assent forms will additionally include the interviewer names, the date/time of consent/assent, the language of consent/assent, and whether others (witnesses) were present. All consent/assent forms will be available in English and translated into country specific local languages in paper copy. A facilitator trained in Good Clinical Practice and Human Subjects Protection will obtain the informed consent from each in a private, confidential area after he/she has had the opportunity to ask the facilitator questions.

Participants unable to read or write will use fingerprints in lieu of signature, and a signature will be obtained from literate independent adult witness to confirm the consent process. All relevant information will be provided in both oral and written form in a way that is understandable to the participant. Ample time and opportunity will be given for participants who want to inquire about details of the study. Potential participants will be excluded from participation based on an inability to provide written informed consent. Participants will be offered a copy of the signed informed consent form.

At month six follow up visit, participants will also be asked to complete an addendum to their original consent/assent form regarding changes made to the study since its launch, including an additional 6 months study extension, the impact of COVID-19 on study operations

and additional questions aimed at understanding the impact of COVID-19. The addendum will remind the participant about key parts of the original consent process (e.g. voluntary participation, sensitivity of questions, storage of data) before clarifying the changes made to the study and additional permission being requested. For AGYW < 18 years old, we will contact the AGYW's caregiver to obtain consent (either in person or via telephone) for the changes to the study activities before requesting assent from the AGYW. AGYW 18+ years and parents/caregivers enrolled in the study will provide their own consent.

When the 6-month survey is administered in person, the procedures for the consent addendum will follow those described above. The addendum will request permission for: 1) being offered clinical assessments (HTC, STI testing, and PrEP) during the 6-month follow-up visit; 2) completing a survey (either in person or via telephone) and a clinical assessment in person at 12 months; and 3) receiving reimbursement through a secure system (e.g. e-wallet) if the 12-month survey is completed via telephone.

When the 6-month survey is administered via telephone, the addendum will request permission for: 1) completing the 6-month survey via telephone and the 6-month clinical assessment on site; 2) completing another follow-up visit—including a survey (either in person or via telephone) and a clinical assessment—at 12 months; and 3) receiving reimbursement through a secure system if a survey is completed via telephone. Study staff will record the participant's name and whether the participant has verbally agreed to participate, in addition to the staff member's own name, the date, and the time, electronically in a fillable form stored on a secure server or through a secure online server (e.g. Qualtrics). Participants will receive a copy of the addendum when they are on site.

Documentation of informed consent process will be completed by study staff and filed with the original informed consent form. These documents will all be stored in the requisite regulatory file, and will be available for review by Good Clinical Practice (GCP) monitors and the Project Leadership team. If informed consent is not documented, this would constitute a protocol violation reportable to the protocol team, regulatory bodies and data on such participants would not be included in the analysis.

### *3.2. Risk/Benefits to participants*

**Benefits/risks to participants:** We anticipate that this study will provide immediate benefits to the participants through participating in sessions, testing for HIV/STIs and receiving treatment. Participants will also benefit by being offered PrEP hence preventing HIV infection.

**Potential Risks.**

This research includes a number of potential risks which we outline below. We detail procedures to minimize these risks.

- ❑ *Biological specimen collection*. We have carefully considered availability of procedures for age-appropriate biologically verified HIV and STIs. We have sought out the latest available but least invasive methods based on preparatory research of available test kits, assays, lap space, and pricing. Our team is well trained to handle all testing procedures and complete referrals where necessary.
- ❑ Interested AGYW and FC will provide urine for STI testing, saliva/finger prick for HIV testing, and blood draws for PrEP at all three time points. AGYW and FC who take up PrEP will also be invited to provide a dried blood spot to test for adherence to PrEP at

follow-up visits. Consistent with our previous studies, we will provide participants with urine collection containers and escort them to a private, secure room in which to produce the specimen. We will train staff to use proper procedures to obtain urine from subjects and approved procedures for the safe handling of urine. Staff will decant the urine to centrifuge tubes labeled with unique, anonymous, subject-sample identifiers and store the specimens in refrigerators. We will send our samples to BARC laboratory will use nucleic acid amplification technologies (NAAT) for STI testing and email the results using a password protected server to study staff in the US within one week. AGYW and FC who test positive for an STI will be referred to our DTHF Medical Officers for treatment. We will adhere to all DTHF standard operating procedures for management of STIs and will at minimum include a free single-session directly observed therapy with the following antimicrobials, each of which constitutes a single session therapeutic regimen: N. gonorrhea – Ceftriaxone 250 mg injection (Rocephin®) AND Azithromycin (Zithromax™) 1g orally; C. trachomatis – Azithromycin (Zithromax™) 1g orally; T. vaginalis – Metronidazole (Flagyl™) 2g orally HIV testing and counseling (HTC) will be conducted by DTHF trained and certified staff at the clinical research sites

- ❑ Risks associated with this study concern discomfort or emotional stress related to or resulting from study procedures and a potential breach of confidentiality. Participants who learn they have acquired HIV or STI may experience emotional distress. Participants may feel uncomfortable answering questions or feel uncomfortable with the assessment procedures (e.g., using a computer or tablet, providing a urine sample, saliva/finger prick), and they may get upset when sensitive topics arise during the intervention sessions. Participants may experience discomfort talking to each other about difficult topics during the sessions and feel concerned that other group members will reveal confidential information to outsiders. Unintended disclosure of information contained in medical case files or from HIV testing could lead to social discrimination or actions against participants. The most likely exception to confidentiality is abuse, neglect, age discrepant sexual activity (a <16 year-old reports sex with someone >18 years old), or suicidal ideation or attempts. If a participant reports any of these concerns, we will consult Dr. Atujuna and the on-site DTHF staff member who will ensure appropriate referrals and reporting per DTHF standard operating procedures. Strategies to minimize these risks are described below.

  1) Protection Against a Breach of Confidentiality: Ethical obligations are complicated in studies involving high-risk youth, because researchers often detect previously unidentified psychopathology, abuse, addictions and criminal activities. Ethicists debate whether or not researchers have a moral duty to routinely report such behaviors and to help subjects. However, professional and federal guidelines recognize that risk-related data should be kept confidential so that the adolescent is not placed in jeopardy. These guidelines recognize that routinely reporting all risky behaviors can have stressful or harmful consequences for subjects, discourage them from seeking help, and discourage them from participating in the research. Youth have an ambiguous status in terms of their decisional capabilities. Thus, designing reporting procedures require researchers to weigh the conflict between the principles of respect and beneficence. Our procedures will accommodate our fiduciary responsibility to protect our subjects' autonomy and welfare, respect their expectations for

confidentiality, and yet produce reliable information. We will take several steps to prevent a breach of confidentiality:

(a) We will inform study participants of the limits of confidentiality during the consent and assent process and at each intervention session. Specifically, we will warn participants 1) that South African law mandates reporting of abuse and/or neglect of children, including sexual activity between a 15-year-old and a partner 18 years or older, and 2) that threat of harm to self or others requires intervention by our staff. We will inform participants that criminal behavior, i.e. drug use, is not reported to authorities. Additional protection for children is presented in later sections.

(b) To protect the integrity of participant data, all participants will receive a random code number. This number will be used on all information collected from participants, including questionnaires and biological specimens. We will maintain lists of participants with links between identifying information and code numbers in password-protected or locked files, and only the South African Co-PI, study coordinator, and data managers (not the US team) will have access to these lists. Other study personnel will have access to individual participants' names and code numbers on an as-needed basis in order to adequately perform their duties, i.e., interviewers must label the questionnaires with the correct code number of the participant.

(c) We will follow extensive data security procedures. We will store hard copies of data in locked cabinets at DTHF and only study staff will have access. After each assessment, data with code numbers will be placed in a locked file cabinet for data entry. Data will be stored on a subdirectory of a server, maintained by DTHF, University of Cape Town, and/or UIC. Only study staff will have access to this subdirectory. UIC will have access to coded elements of dates (i.e. date of birth and medical dates of service), which will be used to calculate windows of time/age that will be used for coded dataset analyses. UIC will not have access to any direct or other participant identifiers, nor to the DTHF master list or medical record which could more directly identify the data. A secure server (e.g. Share point, Box) will be used to store data and to transfer data between DTHF and UIC. The Co-PIs will review all external requests to use the data, and data files provided to individuals will be stripped of identifiers. Files will contain code numbers so that data across assessment waves can be matched. Research assistants with access to the data will be monitored closely and informed about the importance of confidentiality when they join the project and again at laboratory meetings throughout the study. We will prepare written reports without identifiable information about specific participants. When the study ends, the master list will be destroyed, thus removing any link between participants and their data.

(d) The importance of confidentiality will be reviewed at the start of the intervention workshop. We will review group expectations/rules, including the importance of confidentiality. Group members will be reminded throughout the program that what is said in the group is confidential. We have not experienced any adverse events related to breaches of confidentiality in any of our previous intervention studies. To preserve confidentiality during HIV testing, preliminary anonymous HTC will be done. Following DTHF protocols, we will assign an anonymous identifier from personal information. This procedure protects individuals and enables them to

recreate the identifier to obtain test results or when being re-tested. This procedure also enables our team to distinguish between new and repeat testing while preserving anonymity. If a participant tests positive for HIV she will be referred for treatment and care at DTHF and we will assist in transportation to the appointment as needed.

(e) We will maintain confidentiality between FC and AGYW. Although FC and AGYW will be participating jointly in the research study, we will conduct all surveys and interviews separately – FC will not have access to AGYW's confidential responses and vice versa. In addition, we will not share with FC the AGYW's health-related test results, per South Africa's Children's Act 38 of 2005, which 1) allows children to provide independent informed consent for medical treatment if they are at least 12 years old and demonstrate sufficient maturity to understand and provide consent, and 2) mandates that every child has a right to confidentiality regarding his or her health status. Even though we will not disclose AGYW's status to their FC, we recognize that it may be in the best interests of an HIV-positive adolescent to disclose her status to a parent or other trusted adult, given the nature of HIV as a chronic disease that requires on-going support. When appropriate, we will encourage HIV positive AGYW to disclose their status to a trusted adult, and we will offer support and assistance to AGYW concerned about the effect of such a disclosure. DTHF has used these reporting and disclosure procedures with adolescents for many years without adverse effects. During the consent/assent process with AGYW, we will inform them that although we will not share their results with their FC, FC will know they were tested for STIs and may ask them directly about their results. In order to notify AGYW privately from FC and to keep their results confidential, we will document AGYW's preferred contact methods and information, and we will ask AGYW to create a unique passcode that we will use to confirm her identity before providing test results. We will also make clear to FC during the consent process that we will not disclose confidential information provided by AGYW, including test results. Co-PIs have used these procedures for many years with no adverse effects.

**Protection Against Discomfort. We will follow several procedures to minimize participant discomfort.** (1) We will tell participants that their involvement in the study is completely voluntary, and that if any part of the procedure leads them to feel uncomfortable or uneasy they are free to discontinue it. They need only tell study staff, interventionists, or interviewers that they do not want to participate. (2) We will provide a palm card containing information on how to contact study staff to report adverse events related to the study, including emotional distress. (3) All DTHF staff who conduct HTC will be trained, certified, and highly experience with the process involving youth and adults. Pre-test HIV counseling anticipates and helps prepare participants for the possibility of receiving results indicating they are positive. Post-test counseling focuses on the response to the results and planning for treatment if positive or maintaining safe behaviors if negative. We will assist in scheduling an appointment and help transport participants as needed. (4) In the event that someone experiences considerable distress as a result of the procedures, or discloses abuse, neglect, age discrepant sexual activity, or suicidal ideation or intent, we will contact Dr. Atujuna and the onsite staff including a social worker to evaluate her for potential imminent risk. As a research organization, we abide by a detailed manual that outlines how to evaluate individuals for imminent risk and the proper follow-up procedures (e.g., telephone the police, escort to a hospital). DTHF Medical Officers will be on-call for any problems that may arise during the

interviews or intervention. These procedures have been in place for many years to address crisis situations, including consultation with an on-call Medical Officers at DTHF. We will also distribute a "Treatment Resources List" to all participants that includes addresses and phone numbers where mental health and substance abuse services may be obtained. Encouraging self-referrals is appropriate in situations where the risk to the participant is not life-threatening or potentially dangerous. Studies show that participants appreciate information on where to seek help.

**Additional Protections for Children.** We put into place additional protections for children since adolescents who are 15-17 years of age will be in this study. We recognize that children may be a vulnerable group, and extreme care is required to ensure protection and empowerment amongst participants but that exclusion of this group would significantly prohibit scientific development in topic areas of great importance to the health and wellbeing of this group. To ensure informed consent and assent, we will clarify what information will be kept confidential and what will be disclosed to another party. We also build upon our team's extensive research and clinical experience working with adolescents affected by HIV, STIs, and poor mental health in South Africa as well as our team's experience conducting bio-behavioral research with vulnerable populations affected by HIV in South Africa and other international settings. We provide additional protections in consent and assent procedures. All informed assent forms will be read aloud in participants' chosen language and participants will also be provided copies. To ensure that children do not feel obliged to participate in the research, emphasis will be placed on their ability to refuse to participate, or to cease participation at any point during the research. As has been the practice in our previous studies with this vulnerable population, our research team is trained to recognize that any avoidance by children of the research will be taken as evidence of failure to assent. For adolescents, during the parental informed consent procedures and during the adolescent informed assent procedures, we emphasize that all information shared with us will remain confidential except for certain information requiring their permission to disclose (HIV, STI, and other mental health); we will also detail mandated disclosures in the case of harm to self or others, age-differential partners, any sexual acts between a minor under the age of 16 years with an adult who is over 16, perpetration with identifiable rape victims, being a victim of rape, sexual abuse or physical abuse which falls under legally mandated reporting to police, social services, and IRB.

### *3.3 Privacy, data, confidentiality:*

All study activities (study preparation, data collection and analysis, and dissemination of findings) will adhere to strict procedures for protecting and maintaining all participants' privacy and confidentiality as described above. Only PIs, significant contributors, and other essential project staff will have access to project data. All data will be protected by unique research identification numbers (PTIDs). Identifiable data will be kept separate from documents containing other participant data. Paper documents relating to patient data will be kept in locked cabinets accessible only to essential study personnel. Data will also be backed up by the data enterer onto an encrypted hard-drive on a weekly basis and transferred via two-way encryption via the nCrypted Cloud program for PIs and co-I to

oversee for quality control (e.g., an external hard drive, which will be kept in a locked project office). Participants' names will never appear in any report resulting from the project.

### *3.4. Data Protections*

We will follow extensive data security procedures. We will store hard copies of data in locked cabinets at DTHF and only study staff will have access. *First*, all participants will be given participant ID (PTID) and after each assessment, data will be identified using a PTID. *A*ll data will be stored on password-protected computers including smartphones and files. *Second*, all files on project computers and phones be further protected by nCrypted Cloud software which offers two-way encryption with secure access controlled by PIs (who can turn on and off access to password protected files from a central location) and wipe all data from devices remotely in the case of staff leaving the project or in the unlikely event of theft of devices. NCrypted Cloud also enables the PIs to control who has access, who can move files from the secured and encrypted cloud serve onto local hard drives (including computers, phones, and external hard drives), and whether and how files can be moved, providing absolute control over data management and monitoring. *Third*, all staff will be trained in procedures for maintaining confidentiality of participant information. *Fourth*, audiotapes will be delivered via a secure method to transcribers and each tape will be labelled with a code to ensure name confidentiality. Data analyses will only focus on data associated with PTIDs. All other identifiers will be expunged from transcripts. The study team will be trained on all ethical procedures. Files will contain code numbers so that data across assessment waves can be matched. Research assistants with access to the data will be monitored closely and informed about the importance of confidentiality when they join the project and again at laboratory meetings throughout the study. We will prepare written reports without identifiable information about specific participants. When the study ends, the master list will be destroyed, thus removing any link between participants and their data.

### *3.5. Procedures to Monitor Adverse Events*

Adverse events that are anticipated include the need to violate participant confidentiality, inadvertent breach of confidentiality, and participant distress. We will provide participants with a palm size card containing information on how to contact the study staff to report adverse events related to the study, including emotional distress. Study personnel will be trained regarding the limits of confidentiality. This training will include reviewing possible scenarios and knowledge of key questions to assess risk. We will train staff to err on the side of caution and to contact Dr. Atujuna and the on-site DTHF staff as needed. Medical Officers at DTHF will be available by phone 24- hours a day should staff need to consult regarding an emergency. In this situation, we will train staff members to leave participants in the company of study personnel and immediately contact DTHF trained staff before participants leave. Adverse events that are unanticipated will be brought to the attention of the Co-PIs and reported immediately to the Institutional IRB. The IRB at each institution will determine whether it is appropriate to stop the study protocol temporarily or provide suggestions/modifications to the study procedures. Possible modifications include adding adverse events to the consent documents and re-consenting all study participants. The SA and US study coordinators and Dr. Atujuna will be responsible for monitoring participant safety on a monthly basis at regularly

scheduled research meetings. The study coordinators will keep a written log of all adverse events and ensure that the various IRB are contacted immediately. The study coordinators will also keep a log of the outcome of IRB decisions regarding adverse events and apprise the research team of any changes that need to occur as a result.

### 3.6 Policy on Data Sharing

PIs and co-PIs at each study site will control all rights to the data and intellectual property obtained from this study. UIC collaborators will have access to indirect identifiers or coded elements of dates (i.e. date of birth and medical dates of service). All data that are transferred between investigators will be done securely through a secure server (e.g. Share point, Box). To ensure confidentiality, data shared with external investigators or others to replicate or review findings will be blinded of any identifying participant information.

### 3.7 Publication policy

A whole or part of this trial results will be communicated, orally presented, and/or published in appropriate scientific journals. Full anonymity of participant's details will be maintained throughout. Participants wanting to see the results of the trial can request a copy of the article from the investigators once it has been published.

### 3.6 Study timelines

| | Year 1 (Sept 2018-Aug 2019) | | | | | | | | | | | | Year 2 (Sept 2019-Aug 2020) | | | | | | | | | | | | Year 3 (Sept 2020-) | | | | | |
|---|---|---|---|---|---|---|---|---|---|---|---|---|---|---|---|---|---|---|---|---|---|---|---|---|---|---|---|---|---|---|
| **Month** | 9 | 10 | 11 | 12 | 1 | 2 | 3 | 4 | 5 | 6 | 7 | 8 | 9 | 10 | 11 | 12 | 1 | 2 | 3 | 4 | 5 | 6 | 7 | 8 | 9 | 10 | 11 | 12 | 1 | 2 |
| **Aim 1** | | | | | | | | | | | | | | | | | | | | | | | | | | | | | | |
| Study setup, creation of Community Advisory Board, Ethics submission | ■ | ■ | ■ | ■ | ■ | ■ | | | | | | | | | | | | | | | | | | | | | | | | |
| Staff training | | | | | | | ■ | | | ■ | | | ■ | | | | | | | | | | | | | | | | | |
| Participant recruitment for theater testing | | | | | | | | | | ■ | ■ | | | | | | | | | | | | | | | | | | | |
| Theater testing and analysis of findings | | | | | | | | ■ | ■ | ■ | ■ | ■ | | | | | | | | | | | | | | | | | | |
| Finalization of materials, procedures, tools, instruments | | | | | | | | | | | | | ■ | | | | | | | | | | | | | | | | | |
| **Aim 2** | | | | | | | | | | | | | | | | | | | | | | | | | | | | | | |
| Participant recruitment for pilot study | | | | | | | | | | | | ■ | ■ | ■ | ■ | | | | | | | | | | | | | | | |
| Pilot study (incl. baseline surveys, intervention, and clinical testing) | | | | | | | | | | | | | | ■ | ■ | ■ | ■ | ■ | | | | | | | | | | | | |
| Ethics amendment submission | | | | | | | | | | | | | | | | | | | ■ | ■ | ■ | | | | | | | | | |
| 6 months follow-up | | | | | | | | | | | | | | | | | | | | | | ■ | ■ | ■ | | | | | | |
| 12 months follow-up | | | | | | | | | | | | | | | | | | | | | | ■ | ■ | ■ | | ■ | ■ | ■ | ■ | ■ |
| Preparation for randomized controlled trial | | | | | | | | | | | | | | | | | | | | ■ | ■ | ■ | ■ | ■ | ■ | ■ | | | | |

## REFERENCES


1. Abdool Karim Q, Dellar R. Inclusion of adolescent girls in HIV prevention research - an imperative for an AIDS-free generation. J Int AIDS Soc. 2014;17:19075.
2. World Health Organization. Consolidated guidelines on HIV prevention, diagnosis, treatment and care for key populations. Geneva: World Health Organization; 2014.
3. Joint United Nations Programme on HIV/AIDS (UNAIDS). The Gap Report. Geneva: UNAIDS; 2014.
4. Shisana O, Rehle T, Simbayi L, Zuma K, Jooste S, Zungu N, et al. South African National HIV Prevalence, Incidence and Behaviour Survey, 2012. Cape Town: HSRC Press; 2014.
5. Cohen MS. Classical sexually transmitted diseases drive the spread of HIV-1: back to the future. J Infect Dis. 2012;206.
6. Wilton J. STIs: What role do they play in HIV transmission? : CATIE; 2012 [Available from: http://www.catie.ca/en/pif/spring-2012/stis-what-role-do-they-play-hiv-transmission.
7. Johnson LF, Coetzee DJ, Dorrington RE. Sentinel surveillance of sexually transmitted infections in South Africa: a review. Sex Transm Infect. 2005;81(4):287-93.
8. Naidoo S, Wand H, Abbai NS, Ramjee G. High prevalence and incidence of sexually transmitted infections among women living in Kwazulu-Natal, South Africa. AIDS Res Ther. 2014;11(1):31.
9. O'Leary A, Jemmott JB, 3rd, Jemmott LS, Teitelman A, Heeren GA, Ngwane Z, et al. Associations between psychosocial factors and incidence of sexually transmitted disease among South African adolescents. Sex Transm Dis. 2015;42(3):135-9.
10. UNICEF. Towards an AIDS-free Generation: Children and AIDS Sixth Stocktaking Report. New York, NY: UNICEF; 2013.
11. World Health Organization. Adolescents: health risks and solutions 2016 [Available from: http://www.who.int/mediacentre/factsheets/fs345/en/.
12. Shisana O, Rehle T, Simbayi LC, Zuma K, Jooste S, Pillay-van-Wyk V, et al. South African National HIV Prevalence, Incidence, Behaviour and Communication Survey 2008: A Turning Tide Among Teenagers? Cape Town, South Africa: HSRC Press; 2009.
13. Gupta GR. How men's power over women fuels the HIV epidemic. BMJ. 2002;324(7331):183-4.
14. Peltzer K, Pengpid S. Sexuality of 16- to 17- years old South Africans in the context of HIV/AIDS. Soc Behav Pers. 2006;34:239-56.
15. Eaton L, Flisher A, Aaro L. Unsafe sexual behavior among South African youth. Soc Sci Med. 2003;56:149-65.
16. Joint United Nations Programme on HIV/AIDS (UNAIDS). UNAIDS STRATEGY 2016-2021: On the Fast-Track to End AIDS. Geneva: UNAIDS; 2015.

17. Rohleder P, Swartz L, Kalichman S, Simbayi L. Introduction and Overview. In: Rohleder P, Swartz L, Kalichman S, Simbayi L, editors. HIV/AIDS in South Africa 25 Years On: Psychosocial Perpectives. New York: Springer; 2009. p. 1-12.
18. Coovadia H, Jewkes R, Barron P, Sanders D, McIntyre D. The health and health system of South Africa: historical roots of current public health challenges. Lancet. 2009;374(9692):817-34.
19. Karim SS, Churchyard GJ, Abdool KQ, Lawn SD. HIV infection and tuberculosis in South Africa: an urgent need to escalate the public health response. Lancet. 2009;374:921-33.
20. Watts C, Seeley J. Addressing gender inequality and intimate partner violence as critical barriers to an effective HIV response in sub-Saharan Africa. J Int AIDS Soc. 2014;17:19849.
21. Bertrand J, O'Reilly K, Denison J, Anhang R, Sweat M. Systematic review of the effectiveness of mass communication programs to change HIV/AIDS-related behaviors in developing countries. Health Educ Res. 2006;21(4):567-97.
22. Earls F, Raviola G, Carlson M. Promoting child and adolescent mental health in the context of the HIV/AIDS pandemic with a focus on sub-Saharan Africa. J Child Psychol Psychiatry. 2008;49:295-312.
23. Flisher AJ, Mukoma W, Louw J. Evaluating adolescent sexual and reproductive health interventions in Southern and Eastern Africa. In: Klepp K-I, Flisher AJ, S K, editors. Promoting Adolescent Sexual and Reproductive Health in Eastern and Southern Africa. Uppsala, Sweden: Nordic Africa Institute; 2007.
24. Kaaya SF, Mukoma W, Flisher AJ, Klepp K-I. School-based sexual health interventions in Sub-Saharan Africa: A review. Social Dynamics 2002;28:64 – 88.
25. Mukomo W, Flisher A. A systematic review of school-based HIV prevention programs in South Africa. In: Klepp K, Flisher AJ, Kaaya S, editors. Promoting Sexual and Reproductive Health in East and Southern Africa. Pretoria: HSRC Press; 2008. p. 267-87.
26. Gupta GR, Parkhurst JO, Ogden JA, Aggleton P, Mahal A. Structural approaches to HIV prevention. Lancet. 2008;372(9640):764-75.
27. Jemmott LS, Jemmott JB, 3rd, Ngwane Z, Icard L, O'Leary A, Gueits L, et al. 'Let Us Protect Our Future' a culturally congruent evidenced-based HIV/STD risk-reduction intervention for young South African adolescents. Health Educ Res. 2014;29(1):166-81.
28. Pettifor A, Rees HV, Kleinschmidt, et al. Young people's sexual health in South Africa: HIV prevelance and sexual behaviors from a nationally representative household survey. AIDS. 2005;19:1525-34.
29. Blankenship KM, Smoyer AB. Between spaces: Understanding movement to and from prison as an HIV risk factor.  Crime, HIV and health: Intersections of criminal justice and public health concerns: Springer; 2013. p. 207-21.
30. Coates TJ, Richter L, Caceres C. Behavioural strategies to reduce HIV transmission: how to make them work better. Lancet. 2008;372(9639):669-84.
31. Abdool Karim Q, Meyer-Weitz A, Harrison A. Interventions with youth in high prevalence areas. In: Mayer KH, Pizer HF, editors. HIV prevention: A comprehensive approach. London: Academic Press; 2009. p. 407-43.
32. Brawner BM. A Multilevel Understanding of HIV/AIDS Disease Burden among African American Women. Journal of obstetric, gynecologic, and neonatal nursing : JOGNN / NAACOG. 2014;43(5):633-E50.
33. Jackson C, Geddes R, Haw S, Frank J. Interventions to prevent substance use and risky sexual behaviour in young people: a systematic review. Addiction. 2012;107(4):733-47.

34. Dietrich J, Sikkema K, Otwombe KN, Sanchez A, Nkala B, de Bruyn G, et al. Multiple levels of influence in predicting sexual activity and condom use among adolescents in Soweto, Johannesburg, South Africa. J HIV AIDS Soc Serv. 2013;12(3-4):404-23.
35. Auerbach JD, Parkhurst JO, Caceres CF. Addressing social drivers of HIV/AIDS for the long-term response: conceptual and methodological considerations. Glob Public Health. 2011;6 Suppl 3:S293-309.
36. Baird SJ, Garfein RS, McIntosh CT, Ozler B. Effect of a cash transfer programme for schooling on prevalence of HIV and herpes simplex type 2 in Malawi: a cluster randomised trial. Lancet. 2012;379(9823):1320-9.
37. Merson MH, O'Malley J, Serwadda D, Apisuk C. The history and challenge of HIV prevention. Lancet. 2008;372(9637):475-88.
38. Chandra-Mouli V, Lane C, Wong S. What Does Not Work in Adolescent Sexual and Reproductive Health: A Review of Evidence on Interventions Commonly Accepted as Best Practices. Glob Health Sci Pract. 2015;3(3):333-40.
39. Hankins CA, de Zalduondo BO. Combination prevention: a deeper understanding of effective HIV prevention. AIDS. 2010;24 Suppl 4:S70-80.
40. Kurth AE, Celum C, Baeten JM, Vermund SH, Wasserheit JN. Combination HIV prevention: significance, challenges, and opportunities. Curr HIV/AIDS Rep. 2011;8(1):62-72.
41. Folkers GK, Fauci AS. Controlling and ultimately ending the HIV/AIDS pandemic: a feasible goal. JAMA. 2010;304(3):350-1.
42. Padian NS, McCoy SI, Karim SS, Hasen N, Kim J, Bartos M, et al. HIV prevention transformed: the new prevention research agenda. Lancet. 2011;378(9787):269-78.
43. Anderson K, Beutel A, Maughan-Brown B. HIV risk perceptions and first sexual intercourse among youth in Cape Town, South Africa. Int Fam Plan Perspect. 2007;33:98-105.
44. MacPhail C, Campbell C. 'I think condoms are good but, aai, I hate those things': Condom use among adolescents and young people in a Southern African township. Soc Sci Med. 2001;52(11):1613-27.
45. Peltzer K, Promtussananon S. HIV/AIDS knowledge and sexual behavior among junior secondary school students in South Africa. Journal of Social Sciences. 2005;1:1-8.
46. Simbayi L, Kalichman S, Jooste S, Cherry C, Mfecane S, Cain D. Risk factors for HIV-AIDS among youth in Cape Town, South Africa. AIDS Behav. 2005;9:53-61.
47. Hargreaves J, Morison L, Kim J, Bonell C, Porter J, Watts C, et al. The association between school attendance, HIV infection, and sexual behavior among young people in rural South Africa. Journal of Epidemiology Community Health. 2008;62:113-9.
48. Karnell A, Cupp P, Zimmerman R, Feist-Price S, Bennie T. Efficacy of an American alcohol and HIV prevention curriculum adapted for use in South Africa: Results of pilot study in five township schools. AIDS Educ Prev. 2006;18:295-310.
49. Donenberg GR, Pao M. Youths and HIV/AIDS: Psychiatry's role in a changing epidemic. J Am Acad Child Adolesc Psychiatry. 2005;44(8):728-47.
50. Collins P, Freeman M. Bridging the gap between HIV and mental health services in South Africa. In: Rohleder P, Swartz L, Kalichman S, Simbayi L, editors. HIV/AIDS in South Africa 25 Years On: Psychosocial Perspectives. New York: Springer; 2009. p. 353-71.
51. Donenberg GR, Emerson E, Mackesy-Amiti. Sexual risk among African American girls: Psychopathology and mother-daughter relationships. J Consult Clin Psychol. 2011;79(2):153-8.

52. Starr LR, Donenberg GR, Emerson E. Bidirectional Linkages between Psychological Symptoms and Sexual Activities among African- American Adolescent Girls in Psychiatric Care. Journal of clinical child and adolescent psychology : the official journal for the Society of Clinical Child and Adolescent Psychology, American Psychological Association, Division 53. 2012;41(6):811-21.
53. Mutumba M, Harper GW. Mental health and support among young key populations: an ecological approach to understanding and intervention2015.
54. Stein D, Seedat S, Herman A, et al. Lifetime prevalence of psychiatric disorders in South Africa. Br J Psychiatry. 2008;192:112-7.
55. Kessler RC, Berglund P, Demler O, Jin R, Merikangas KR, Walters EE. Lifetime prevalence and age-of-onset distributions of DSM-IV disorders in the National Comorbidity Survey Replication. Arch Gen Psychiatry. 2005;62(6):593-602.
56. Merikangas KR, He JP, Burstein M, Swanson SA, Avenevoli S, Cui L, et al. Lifetime prevalence of mental disorders in U.S. adolescents: results from the National Comorbidity Survey Replication--Adolescent Supplement (NCS-A). J Am Acad Child Adolesc Psychiatry. 2010;49(10):980-9.
57. Brook D, Morojele N, Zhang C, Brook J. South African adolescents: Pathways to risky sexual behavior. AIDS Educ Prev. 2006;18:259-72.
58. Bhana A, Peterson I. HIV and Youth: A Behavioral Perspective. In: Rohleder P, Swartz L, Kalichman S, Simbayi L, editors. HIV/AIDS in South Africa 25 Years On: Psychosocial Perspectives. New York: Springer; 2009. p. 55-68.
59. Freeman M, Patel V, Collins P, Bertolote J. Integrating mental health in global initiatives for HIV/AIDS. Br J Psychiatry. 2005;187:1-3.
60. Patel V, Flisher A, Hetrick S, McGorry P. Mental health of young people: A global public health challenge. Lancet. 2007;369:1302-13.
61. Friedman SR, Aral S. Social networks, risk-potential networks, health, and disease. J Urban Health. 2001;78(3):411-8.
62. Youm Y, Laumann EO. Social network effects on the transmission of sexually transmitted diseases. Sex Transm Dis. 2002;29(11):689-97.
63. Millett GA, Peterson JL, Wolitski RJ, Stall R. Greater risk for HIV infection of black men who have sex with men: a critical literature review. Am J Public Health. 2006;96(6):1007-19.
64. Blaser N, Wettstein C, Estill J, Vizcaya LS, Wandeler G, Egger M, et al. Impact of viral load and the duration of primary infection on HIV transmission: systematic review and meta-analysis. AIDS. 2014;28(7):1021-9.
65. Grieb SM, Davey-Rothwell M, Latkin CA. Concurrent sexual partnerships among urban African American high-risk women with main sex partners. AIDS Behav. 2012;16(2):323-33.
66. Emerson E, Donenberg GR, Wilson HW. Health-protective effects of attachment among African American girls in psychiatric care. J Fam Psychol. 2012;26(1):124-32.
67. Jaccard J, Dittus PJ, Gordon VV. Parent-adolescent congruency in reports of adolescents sexual behavior and in communications about sexual behavior. Child Dev. 1998;69(1):247-61.
68. Aronowitz T, Rennells RE, Todd E. Heterosocial behaviors in early adolescent African American girls: The role of mother-daughter relationships. J Fam Nurs. 2005;11(2):122-39.

69. O'Donnell L, Stueve A, Durnan R, Myint-U A, Agronick G, San Doval A, et al. Parenting practices, parents' underestimation of daughters' risks, and alcohol and sexual behaviors of urban girls J Adolesc Health. 2008;42:496-502.
70. Meschke LL, Bartholomae S, Zentall SR. Adolescent sexuality and parent-adolescent processes: Promoting healthy teen choices. J Adolesc Health. 2002;31(6S):264-79.
71. Dutra R, Miller KS, Forehand R. The process and content of sexual communication with adolescents in two-parent families: Associations with sexual risk-taking behavior. AIDS Behav. 1999;3(1):59-66.
72. Miller KS, Forehand R, Kotchick BA. Adolescent sexual behavior in two ethnic minority samples: The role of family variables. Journal of Marriage and the Family. 1999;61(2):85-98.
73. Whitaker DJ, Miller KS, May DC, Levin ML. Teenage partners' communication about sexual risk and condom use: The importance of parent-teenager discussions. Fam Plann Perspect. 1999;31(3):117-21.
74. Miller KS, Fasula A, Dittus P, Wiegand R, Wyckoff S, McNair L. Barriers and facilitators to maternal communication with preadolescents about age-relevant sexual topics. AIDS Behav. 2007.
75. Babalola S, Tambashe BO, Vondrasek C. Parental factors and sexual risk-taking among young people of Cote d'Iviore. Afr J Reprod Health. 2005;9:49-65.
76. Poulsen M, Miller K, Lin C, Fasula A, Vandenhoudt H, Wyckoff S, et al. Factors associated with parent-child communication about HIV/AIDS in the United States and Kenya: A cross-cultural comparison. AIDS Behav. 2009.
77. Adu-Mireku S. Family communication about HIV/AIDS and sexual behavior among senior secondary school students in Accra, Ghana. Afr Health Sci. 2003;3:7-14.
78. Crichton J, Ibisomi L, Gyimah SO. Mother-daughter communication about sexual maturation, abstinence and unintended pregnancy: experiences from an informal settlement in Nairobi, Kenya. J Adolesc. 2012;35(1):21-30.
79. Juma M, Alaii J, Askew A, Bartholomew L, Borne B. Community Perspectives on Parental/Caregiver Communication on Reproductive Health and HIV with Adolescent Orphans and Non-Orphans in Western Kenya. J Child Adolesc Behav. 2015;3(206).
80. Soon CN, Kaida A, Nkala B, Dietrich J, Cescon A, Gray G, et al. Adolescent experiences of HIV and sexual health communication with parents and caregivers in Soweto, South Africa. SAHARA J. 2013;10(3-4):163-9.
81. Dimbuene ZT, Defo BK. Risky sexual behaviour among unmarried young people in Cameroon: another look at family environment. J Biosoc Sci. 2011;43(2):129-53.
82. Puffer ES, Meade CS, Drabkin AS, Broverman SA, Ogwang-Odhiambo RA, Sikkema KJ. Individual- and family-level psychosocial correlates of HIV risk behavior among youth in rural Kenya. AIDS Behav. 2011;15(6):1264-74.
83. Chopra M, Lawn J, Sanders D, Barron P, Karim S, Bradshaw D, et al. Achieving the health Millennium Development Goals for South Africa: challenges and priorities. Lancet. 2009;374(9694):1023-31.
84. Brown LK, Hadley W, Donenberg GR, DiClemente RJ, Lescano C, Lang DM, et al. Project STYLE: A Multisite RCT for HIV Prevention Among Youths in Mental Health Treatment. Psychiatr Serv. 2014;65(3):338-44.
85. Harrison A, Newell M-L, Imrie J, Hoddinott G. HIV prevention for South African youth: which interventions work? A systematic review of current evidence. BMC Public Health. 2010;10(1):102.

86. Kuo C, Atujuna M, Mathews C, Stein DJ, Hoare J, Beardslee W, et al. Developing family interventions for adolescent HIV prevention in South Africa. AIDS Care. 2016;28 Suppl 1:106-10.
87. Strathdee SA, Wechsberg WM, Kerrigan DL, Patterson TL. HIV prevention among women in low- and middle-income countries: intervening upon contexts of heightened HIV risk. Annu Rev Public Health. 2013;34:301-16.
88. Joint United Nations Programme on HIV/AIDS. UNAIDS Special Report: How Africa Turned AIDS Around. Geneva: UNAIDS; 2013.
89. Wingood GM, DiClemente RJ. Application of the theory of gender and power to examine HIV-related exposures, risk factors, and effective interventions for women. Health Educ Behav. 2000;27(5):539-65.
90. Wingood GM, DiClemente RJ. Partner influences and gender-related factors associated with noncomdom use among young adult African American women. Am J Community Psychol. 1998;26(1):29-51.
91. Jewkes R. HIV and Women. In: Rohleder P, Swartz L, Kalichman S, Simbayi L, editors. HIV/AIDS in South Africa 25 Years On. New York: Springer; 2009.
92. Leclerc-Madlala, Simbayi L, Cloete A. The Sociocultural Aspects of HIV/AIDS in South Africa. In: Rohleder P, Swartz L, Kalichman S, Simbayi L, editors. HIV/AIDS in South Africa 25 Years On. New York: Springer; 2009.
93. Lindegger G, Quayle. Masculinity & HIV/AIDS. In: Rohleder P, Swartz L, Kalichman S, Simbayi L, editors. HIV/AIDS in South Africa 25 Years On. New York.: Springer; 2009.
94. Prather C, Fuller T, King W, Brown M, Moering M, Little S, et al. Diffusing an HIV prevention intervention for African American women: Integrating Afrocentric components into the SISTA diffusion strategy. AIDS Educ Prev. 2006;18:149-60.
95. Pulerwitz J, Amaro H, De Jong W, Gortmaker SL, Rudd R. Relationship power, condom use and HIV risk among women in the USA. AIDS Care. 2002;14:789-800.
96. Stirling M, Rees H, Kasedde S, Hankins C. Addressing the vulnerability of young women and girls to stop the HIV epidemic in southern Africa. AIDS2008. 2008;22 (Suppl 4):S1-S3.
97. Wood K, Jewkes R. "Dangerous" love: Reflection on violence among Xhosa township youth. In: Morrell R, editor. Changing men in southern Africa. New York: Zed Books Ltd; 2001. p. 317-36.
98. Varga CA. Sexual decision-making and negotiation in the midst of AIDS: youth in KwaZulu/Natal, South Africa. Health Transit Rev. 1997;7:45-67.
99. Jewkes R, Morrell R. Gender and sexuality: emerging perspectives from the heterosexual epidemic in South Africa and implications for HIV risk and prevention. J Int AIDS Soc. 2010;13:6-.
100. Jewkes R, Morrell R. Sexuality and the limits of agency among South African teenage women: theorising femininities and their connections to HIV risk practices. Social science & medicine (1982). 2012;74(11):1729-37.
101. Pettifor AE, Measham DM, Rees HV, Padian NS. Sexual power and HIV risk, South Africa. Emerg Infect Dis. 2004;10(11):1996-2004.
102. Leclerc-Madlala S. Age-disparate and intergenerational sex in southern Africa: the dynamics of hypervulnerability. AIDS. 2008;22 Suppl 4:S17-25.
103. Mah TL. Prevalence and Correlates of Concurrent Sexual Partnerships Among Young People in South Africa. Sex Transm Dis. 2009;36(12).

104. Jipguep M-C, Sanders-Phillips K, Cotton L. Another look at HIV in African American Women: The impact of psychosoical and contextual factors. J Black Psychol. 2004;30(3):366-85.
105. Mah TL, Halperin DT. Concurrent sexual partnerships and the HIV epidemics in Africa: evidence to move forward. AIDS Behav. 2010;14(1):11-6; dicussion 34-7.
106. Gregson S, Nyamukapa CA, Garnett GP, Mason PR, Zhuwau T, Carael M, et al. Sexual mixing patterns and sex-differentials in teenage exposure to HIV infection in rural Zimbabwe. Lancet. 2002;359(9321):1896-903.
107. Pettifor A, Macphail C, Rees H, Cohen M. HIV and sexual behavior among young people: the South African paradox. Sex Transm Dis. 2008;35(10):843-4.
108. Beauclair R, Kassanjee R, Temmerman M, Welte A, Delva W. Age-disparate relationships and implications for STI transmission among young adults in Cape Town, South Africa. Eur J Contracept Reprod Health Care. 2012;17(1):30-9.
109. Cluver L, Boyes M, Orkin M, Pantelic M, Molwena T, Sherr L. Child-focused state cash transfers and adolescent risk of HIV infection in South Africa: a propensity-score-matched case-control study. The Lancet Global Health. 2013;1(6):e362-e70.
110. Shisana O, Rehle T, Simbayi L, et al. South African national HIV prevalence, HIV incidence, behavior and communication survey. Cape Town, South Africa: HSRC Press; 2005.
111. Amaro H, Raj A. On the margin: Power and women's HIV risk reduction strategies. Sex Roles. 2000;42:723-49.
112. Gutierrez LM, Oh HJ, Gillmore MR. Toward an understanding of (Em)Power(Ment) for HIV/AIDS prevention with adolescent women. Sex Roles. 2000;42:681-11.
113. Sanders-Phillips K. Factors influencing HIV/AIDS in women of color. Public Health Rep. 2002;117:S151-S6.
114. Teitelman AM, Ratcliffe SJ, Morales-Aleman MM, Sullivan CM. Sexual relationship power, intimate partner violence, and condom use among minority urban girls. Journal of Interpersonal Violence. 2008;23(12):1694-712.
115. Decker MR, Peitzmeier S, Olumide A, Acharya R, Ojengbede O, Covarrubias L, et al. Prevalence and Health Impact of Intimate Partner Violence and Non-partner Sexual Violence Among Female Adolescents Aged 15-19 Years in Vulnerable Urban Environments: A Multi-Country Study. J Adolesc Health. 2014;55(6 Suppl):S58-67.
116. Mathews C, Aaro L, Flisher A, Mukoma W, Wubs A, Schaalma H. Predictors of first sexual intercourse among adolescents in Cape Town, South Africa. Health Educ Res. 2009;24(1):1-10.
117. Dunkle KL, Jewkes RK, Brown HC, Gray GE, McIntryre JA, Harlow SD. Gender-based violence, relationship power, and risk of HIV infection in women attending antenatal clinics in South Africa. Lancet. 2004;363(9419):1415-21.
118. Jewkes R, Dunkle K, Nduna M, Levin J, Jama N, Khuzwayo N, et al. Factors associated with HIV sero-status in young rural South African women: connections between intimate partner violence and HIV. Int J Epidemiol. 2006;35(6):1461-8.
119. Li Y, Marshall CM, Rees HC, Nunez A, Ezeanolue EE, Ehiri JE. Intimate partner violence and HIV infection among women: a systematic review and meta-analysis. J Int AIDS Soc. 2014;17:18845.
120. Jewkes RK, Dunkle K, Nduna M, Shai N. Intimate partner violence, relationship power inequity, and incidence of HIV infection in young women in South Africa: a cohort study. Lancet. 2010;376(9734):41-8.

121. MacPhail C, Williams BG, Campbell C. Relative risk of HIV infection among young men and women in a South African township. Int J STD AIDS. 2002;13(5):331-42.
122. Kaufman C, Stavrou S. "Bus fare, please": The economics of sex and gifts among adolescents in urban South Africa (No. 166). Washington, DC: Population Council; 2002.
123. Purdie V, Downey G. Rejection sensitivity and adolescent girls' vulnerability to relationship-centered difficulties. Child Maltreatment. 2000;5(4):338-49.
124. Kerrigan D, Andrinopoulos D, Johnson R, Parham P, Thomas T, Ellen J. Staying strong: Gender ideologies among African-American adolescents and the implications for HIV/STI prevention J Sex Res. 2007;44:172 - 80.
125. Perrino T, Fernandez M, Bowen S, Arheart K. Low-Income African American women's attempts to convince their main partner to use condoms. Cultur Divers Ethnic Minor Psychol. 2006;12:70 - 84.
126. Jewkes RK, Levin JB, Penn-Kekana LA. Gender inequalities, intimate partner violence and HIV preventive practices: findings of a South African cross-sectional study. Soc Sci Med. 2003;56(1):125-34.
127. MacPherson EE, Sadalaki J, Njoloma M, Nyongopa V, Nkhwazi L, Mwapasa V, et al. Transactional sex and HIV: understanding the gendered structural drivers of HIV in fishing communities in Southern Malawi. J Int AIDS Soc. 2012;15 Suppl 1:1-9.
128. Hatzenbuehler ML, Phelan JC, Link BG. Stigma as a fundamental cause of population health inequalities. Am J Public Health. 2013;103(5):813-21.
129. Grossman CI, Stangl AL. Editorial: Global action to reduce HIV stigma and discrimination. J Int AIDS Soc. 2013;16(3 Suppl 2):18881.
130. MacPhail CL, Pettifor A, Coates T, Rees H. "You must do the test to know your status": attitudes to HIV voluntary counseling and testing for adolescents among South African youth and parents. Health Educ Behav. 2008;35(1):87-104.
131. Garcia J, Parker C, Parker RG, Wilson PA, Philbin M, Hirsch JS. Psychosocial Implications of Homophobia and HIV Stigma in Social Support Networks: Insights for High-Impact HIV Prevention Among Black Men Who Have Sex With Men. Health Educ Behav. 2016;43(2):217-25.
132. Hosek S, Celum C, Wilson CM, Kapogiannis B, Delany-Moretlwe S, Bekker L-G. Preventing HIV among adolescents with oral PrEP: observations and challenges in the United States and South Africa2016.
133. McDoom MM, Bokhour B, Sullivan M, Drainoni ML. How older black women perceive the effects of stigma and social support on engagement in HIV care. AIDS Patient Care STDS. 2015;29(2):95-101.
134. Parker W, Dalrymple L, Durden E. Communicating beyond AIDS awareness, a manual for South Africa. Report commissioned by the HIV/AIDS and STD Directorate of the Department of Health as part of the 1997/98 Beyond Awareness campaign. Pretoria: Department of Health; 1998.
135. Goldstein S, Japhet G, Usdin S, Scheepers E. Soul City: A sustainable edutainment vehicle facilitating social change. Health Promot J Austr. 2004;15(2):114-20.
136. Dellar RC, Dlamini S, Karim QA. Adolescent girls and young women: key populations for HIV epidemic control. J Int AIDS Soc. 2015;18(2 Suppl 1):19408.
137. Doyle AM, Ross DA, Maganja K, Baisley K, Masesa C, Andreasen A, et al. Long-term biological and behavioural impact of an adolescent sexual health intervention in Tanzania: follow-up survey of the community-based MEMA kwa Vijana Trial. PLoS Med. 2010;7(6):e1000287.

138. Michielsen K, Chersich MF, Luchters S, De Koker P, Van Rossem R, Temmerman M. Effectiveness of HIV prevention for youth in sub-Saharan Africa: systematic review and meta-analysis of randomized and nonrandomized trials. AIDS. 2010;24(8):1193-202.
139. Mavedzenge SN, Luecke E, Ross D. Effectiveness of HIV Prevention, Treatment and Care Interventions Among Adolescents: A Systematic Review of Systematic Reviews. New York: UNICEF; 2013.
140. Parker W, Colvin M, Birdsall K. Live the future: HIV and AIDS scenarios for South Africa 2005-2025: An overview of factors underlying future trends. Johannesburg, South Africa: Centre for AIDS Development, Research, and Evaluation; 2006.
141. Mantell J, Harrison A, Hoffman S, Smit J, Stein Z, Exner T. The Mpondombili Project: Preventing HIV/AIDS and unintended pregnancy among rural South African school going adolescents\. Reproductive Health Matters. 2006;14:113-22.
142. Smith E, Palen L, Caldwell L, Flisher A, Graham J, Mathews C, et al. Substance use and sexual risk prevention in Cape Town, South Africa: An evaluation of the HealthWise program. Prevention Science. 2008;9:311-21.
143. Kirby D, Obasi A, Laris BA. The effectiveness of sex education and HIV education interventions is schools in developing countries. In: Ross DA, Dick B, Ferguson J, editors. Preventing HIV/AIDS in young people: A systematic review of the evidence from developing countries. Geneva: World Health Organization; 2006. p. 103-50.
144. Visser M. HIV/AIDS prevention through peer education and support in secondary schools in South Africa. Journal of Social Aspects of HIV/AIDS. 2007;4:678-94.
145. Magnani R, MacIntyre K, Karim A, Brown L, Hutchinson P. The impact of life skills education on adolescent sexual risk behaviors in KwaZulu-Natal, South Africa. J Adolesc Health. 2005;36:289-304.
146. Speizer I, Magnani R, Colvin C. The effectiveness of adolescent reproductive health interventions in developing countries: A review of the evidence. J Adolesc Health. 2003;33:324-48.
147. Phetla G, Busza J, Hargreaves J, Pronyk P, Kim J, Morison L, et al. “They have opened our mouths”: Increasing women’s skills and motivation for sexual communication with young people in rural South Africa. AIDS Educ Prev. 2008;20:504-18.
148. Paruk Z, Peterson I, Bhana A. Facilitating health-enabling social contexts for youth: Qualitative evaluation of a family-based HIV-prevention pilot programme. Afr J AIDS Res. 2009;8:61-8.
149. Armistead L, Cook S, Skinner D, Toefy Y, Anthony ER, Zimmerman L, et al. Preliminary results from a family-based HIV prevention intervention for South African youth. Health Psychol. 2014;33(7):668-76.
150. Bhana A, Mellins CA, Petersen I, Alicea S, Myeza N, Holst H, et al. The VUKA family program: piloting a family-based psychosocial intervention to promote health and mental health among HIV infected early adolescents in South Africa. AIDS Care. 2014;26(1):1-11.
151. Bhana A, McKay MM, Mellins C, Petersen I, Bell C. Family-based HIV prevention and intervention services for youth living in poverty-affected contexts: the CHAMP model of collaborative, evidence-informed programme development. J Int AIDS Soc. 2010;13(Suppl 2):S8-S.
152. Bogart LM, Skinner D, Thurston IB, Toefy Y, Klein DJ, Hu CH, et al. Let's Talk!, A South African Worksite-Based HIV Prevention Parenting Program. The Journal of adolescent health : official publication of the Society for Adolescent Medicine. 2013;53(5):602-8.

153. Cluver LD, Orkin FM, Yakubovich AR, Sherr L. Combination Social Protection for Reducing HIV-Risk Behavior Among Adolescents in South Africa. J Acquir Immune Defic Syndr. 2016;72(1):96-104.
154. Hardee K, Gay J, Croce-Galis M, Afari-Dwamena NA. What HIV programs work for adolescent girls? J Acquir Immune Defic Syndr. 2014;66 Suppl 2:S176-85.
155. Stevens JW. Smart and Sassy: The Strenghts of Inner-City Black Girls. New York: Oxford University Press; 2002.
156. Mbugua N. Factors inhibiting educated mothers in Kenya from giving meaningful sex-education to their daughters. Soc Sci Med. 2007;64:1079-89.
157. Izugbara C. Notions of sex, sexuality, and relationship among adolescent boys in rural southeastern Nigeria. Sex Education. 2004;4:63-79.
158. Izugbara C. Home-based sexuality education: Nigerian parents discussing sex with their children. Youth & Society. 2008;30:575-600.
159. Blankenship KM, West BS, Kershaw TS, Biradavolu MR. Power, community mobilization, and condom use practices among female sex workers in Andhra Pradesh, India. AIDS. 2008;22 Suppl 5:S109-16.
160. Venter WD, Cowan F, Black V, Rebe K, Bekker L-G. Pre-exposure prophylaxis in Southern Africa: feasible or not? J Int AIDS Soc. 2015;18(4 Suppl 3):19979.
161. Bekker L-G, Johnson L, Wallace M, Hosek S. Building our youth for the future. J Int AIDS Soc. 2015;18(2 Suppl 1):20027.
162. Baeten JM, Donnell D, Ndase P, Mugo NR, Campbell JD, Wangisi J, et al. Antiretroviral prophylaxis for HIV prevention in heterosexual men and women. N Engl J Med. 2012;367(5):399-410.
163. Thigpen MC, Kebaabetswe PM, Paxton LA, Smith DK, Rose CE, Segolodi TM, et al. Antiretroviral preexposure prophylaxis for heterosexual HIV transmission in Botswana. N Engl J Med. 2012;367(5):423-34.
164. Abdool Karim Q, Abdool Karim SS, Frohlich JA, Grobler AC, Baxter C, Mansoor LE, et al. Effectiveness and safety of tenofovir gel, an antiretroviral microbicide, for the prevention of HIV infection in women. Science. 2010;329(5996):1168-74.
165. Fonner VA, Dalglish SL, Kennedy CE, Baggaley R, O'Reilly KR, Koechlin FM, et al. Effectiveness and safety of oral HIV preexposure prophylaxis for all populations. AIDS. 2016;30(12):1973-83.
166. World Health Organization. HIV/AIDS 2016 [Available from: http://www.who.int/mediacentre/factsheets/fs360/en/.
167. PEPFAR. DREAMS Innovation Challenge Fact Sheet. In: PEPFAR, editor. 2016.
168. Auerbach JD, Hoppe TA. Beyond "getting drugs into bodies": social science perspectives on pre-exposure prophylaxis for HIV. J Int AIDS Soc. 2015;18(4 Suppl 3):19983.
169. Baeten JM, Haberer JE, Liu AY, Sista N. Preexposure prophylaxis for HIV prevention: where have we been and where are we going? J Acquir Immune Defic Syndr. 2013;63 Suppl 2:S122-9.
170. Okwundu CI, Uthman OA, Okoromah CA. Antiretroviral pre-exposure prophylaxis (PrEP) for preventing HIV in high-risk individuals. Cochrane Database Syst Rev. 2012(7):CD007189.
171. Grant RM, Lama JR, Anderson PL, McMahan V, Liu AY, Vargas L, et al. Preexposure chemoprophylaxis for HIV prevention in men who have sex with men. N Engl J Med. 2010;363(27):2587-99.

172. Van Damme L, Corneli A, Ahmed K, Agot K, Lombaard J, Kapiga S, et al. Preexposure prophylaxis for HIV infection among African women. N Engl J Med. 2012;367(5):411-22.
173. Marrazzo JM, Ramjee G, Richardson BA, Gomez K, Mgodi N, Nair G, et al. Tenofovir-based preexposure prophylaxis for HIV infection among African women. N Engl J Med. 2015;372(6):509-18.
174. Collins S. Dapivirine PrEP vaginal ring shows only limited PrEP protection against HIV in African women: HIV Treatment Information Base; 2016 [Available from: http://i-base.info/htb/29667.
175. Cottrell ML, Yang KH, Prince HM, Sykes C, White N, Malone S, et al. A Translational Pharmacology Approach to Predicting Outcomes of Preexposure Prophylaxis Against HIV in Men and Women Using Tenofovir Disoproxil Fumarate With or Without Emtricitabine. J Infect Dis. 2016;214(1):55-64.
176. Marrazzo JM, Ramjee G, Nair G, Palanee T, Mkhize B, Nakabiito C, et al. Pre--exposure prophylaxis for HIV in Women: daily oral tenofovir, oral tenofovir/emtricitabine, or vaginal tenofovir gel in the VOICE Study (MTN 003). Atlanta: 20th Conference on Retroviruses and Opportunistic Infections; 2013.
177. Auerbach JD, Kinsky S, Brown G, Charles V. Knowledge, Attitudes, and Likelihood of Pre-Exposure Prophylaxis (PrEP) Use Among US Women at Risk of Acquiring HIV. AIDS Patient Care STDS. 2015;29(2):102-10.
178. Pettifor A, Nguyen NL, Celum C, Cowan FM, Go V, Hightow-Weidman L. Tailored combination prevention packages and PrEP for young key populations. J Int AIDS Soc. 2015;18(2 Suppl 1):19434.
179. Fletcher FE, Fisher C, Buchberg MK, Floyd B, Ehioba A, Donenberg G. “Where Did This [PrEP] Come From?” African American Mother/Daughter Attitudes Towards Adolescent Pre-Exposure Prophylaxis (PrEP) Utilization and Clinical Trial Participation. AIDS Patient Care and STDs. under review.
180. Adimora AA, Schoenbach VJ. Contextual factors and the black-white disparity in heterosexual HIV transmission. Epidemiology. 2002;13(6):707-12.
181. Adimora AA, Schoenbach VJ. Social Determinants of Sexual Networks, Partnership Formation, and Sexually Transmitted Infections. In: Aral SO, Fenton KA, Lipshutz JA, editors. The New Public Health and STD/HIV Prevention: Personal, Public and Health Systems Approaches. New York, NY: Springer New York; 2013. p. 13-31.
182. Kerr J, Jackson T. Stigma, sexual risks, and the war on drugs: Examining drug policy and HIV/AIDS inequities among African Americans using the Drug War HIV/AIDS Inequities Model. Int J Drug Policy. 2016;37:31-41.
183. van der Straten A, Stadler J, Luecke E, Laborde N, Hartmann M, Montgomery ET. Perspectives on use of oral and vaginal antiretrovirals for HIV prevention: the VOICE-C qualitative study in Johannesburg, South Africa. J Int AIDS Soc. 2014;17(3 Suppl 2):19146.
184. van der Straten A, Stadler J, Montgomery E, Hartmann M, Magazi B, Mathebula F, et al. Women's experiences with oral and vaginal pre-exposure prophylaxis: the VOICE-C qualitative study in Johannesburg, South Africa. PLoS One. 2014;9(2):e89118.
185. Bekker L-G, Hughes J, Amico R, Roux S, Hendrix C, Anderson P, et al. HPTN 067/ADAPT Cape Town: A Comparison of Daily and Nondaily PrEP Dosing in African Women [Abstract 978LB].  Conference on Retroviruses and Opportunistic Infections Seattle, Washington2015.

186. Bekker L-G, Slack C, Lee S, Shah S, Kapogiannis B. Ethical issues in adolescent HIV research in resource-limited countries. J Acquir Immune Defic Syndr. 2014;65 Suppl 1:S24-8.
187. Rhodes SD, Foley KL, Zometa CS, Bloom FR. Lay Health Advisor Interventions Among Hispanics/Latinos: A Qualitative Systematic Review. Am J Prev Med. 2007;33(5):418-27.
188. Tolli MV. Effectiveness of peer education interventions for HIV prevention, adolescent pregnancy prevention and sexual health promotion for young people: a systematic review of European studies. Health Educ Res. 2012;27(5):904-13.
189. Creese A, Floyd K, Alban A, Guinness L. Cost-effectiveness of HIV/AIDS interventions in Africa: a systematic review of the evidence. Lancet. 2002;359(9318):1635-43.
190. Galarraga O, Colchero MA, Wamai RG, Bertozzi SM. HIV prevention cost-effectiveness: a systematic review. BMC Public Health. 2009;9 Suppl 1:S5.
191. Ross EL, Cinti SK, Hutton DW. Implementation and Operational Research: A Cost-Effective, Clinically Actionable Strategy for Targeting HIV Preexposure Prophylaxis to High-Risk Men Who Have Sex With Men. J Acquir Immune Defic Syndr. 2016;72(3):e61-7.
192. Jewell BL, Cremin I, Pickles M, Celum C, Baeten JM, Delany-Moretlwe S, et al. Estimating the cost-effectiveness of pre-exposure prophylaxis to reduce HIV-1 and HSV-2 incidence in HIV-serodiscordant couples in South Africa. PLoS One. 2015;10(1):e0115511.
193. Centers for Disease Control and Prevention. Updated Compendium of Evidenced-Based Interventions 2007 [Available from: http://www.cdc.gov/hiv/topics/research/prs/evidence-based-interventions.htm.
194. DiClemente RJ, Wingood GM. A randomized controlled trial of an HIV sexual risk-reduction intervention for young African-American women. J Am Med Assoc. 1995;274(16):1271-6.
195. Wingood GM, DiClemente RJ. Enhancing adoption of evidence-based HIV interventions: Promotion of a suite of HIV prevention interventions for African American women. AIDS Educ Prev. 2006;18(Supplement A):161-70.
196. Collins CE, Whiters DL, Braithwaite R. The Saved SISTA Project: A faith-based HIV prevention program for black woemn in addiction recovery. American Journal of Health Studies. 2007;22(2):76-82.
197. Centers for Disease Control and Prevention. HIV/AIDS among US women: Minority and young women at continuing risk 2000 [Available from: http://www.cdc.gov/hiv/pubs/facts/women.htm.
198. Cornelius J, Moneyham L, LeGrand S. Adaptation of an HIV prevention curriculum for use with older African American women. J Assoc Nurses AIDS Care. 2008;19(1):16 - 27.
199. DiClemente RJ, Wingood GM, Harrington KF, Lang DL, Davies SL, Hook EW, et al. Efficacy of an HIV prevention intervention for African American adolescent girls: A randomized controlled trial. J Am Med Assoc. 2004;292:171-9.
200. DiClemente R. Development and Evaluation of an HIV Risk-Reduction Intervention Tailored for High-Risk African-American Female Adolescents Seeking Treatment at STD Clinics. Arch Pediatr Adolesc Med. in press.
201. Barnabas RV, Celum C. Bending the curve: maximising impact with focused HIV prevention. Lancet. 2014;384(9939):216-7.
202. Bekker L-G. Adolescent PrEP delivery: Opportunities and Challenges. Seattle: HIV/AIDS Network Coordination (HANC); 2016.

203. Donenberg GR, Brown LK, Hadley W, Kapungu C, Lescano C. Family-based HIV-prevention program for adolescents with psychiatric disorders. In: Pequegnat W, Bell C, editors. Families and HIV/AIDS: Culture and contextual issues in prevention and treatment. New York: Springer; 2012.
204. Kelly JA. HIV risk reduction interventions for persons with severe mental illness. Clin Psychol Rev. 1997;17(3):293-309.
205. Otto-Salaj LL, Kelly JA, Stevenson LY. Implementing cognitive-behavioral AIDS/HIV risk reduction group interventions in community mental health settings that serve people with serious mental illness. Psychiatric Rehabilitation Journal. 1998;21(4):394-404.
206. Kaplan HB. Self-attitudes and deviant behavior. Pacific Palisades, CA: Goodyear; 1975.
207. Kelly JA, McAuliffe TL, Sikkema KJ, Murphy DA, Somlai AM, Mulry G, et al. Reduction in risk behavior among adults with severe mental illness who learned to advocate for HIV prevention. Psychiatr Serv. 1997;48(10):1283-8.
208. Casper LM. Does family interaction prevent adolescent pregnancy? Fam Plann Perspect. 1990;22(3):109-14.
209. Carey MP, Schroder KE. Development and psychometric evaluation of the brief HIV Knowledge Questionnaire. AIDS Educ Prev. 2002;14(2):172-82.
210. Mukoma W, Flisher A, J., Helleve A, Aaro LE, Mathews C, Kaaya S, et al. Development and test-retest reliability of a research instrument designed to evaluate school-based HIV/AIDS interventions in South Africa and Tanzania. Scandanavian Journal of Public Health. 2009;37(Supplement 1):7-15.
211. Achenbach T. Manual for the Child Behavior Checklist/ 4-18 and 1991 Profile. Burlington, VT: University of Vermont, Department of Psychiatry; 1991.
212. Achenbach T. Manual for the Youth Self-Report and 1991 Profile: Department of Psychiatry University of Vermont; 1991.
213. Achenbach T, Rescorla L. Manual for the ASEBA adult forms & profiles. University of Vermont, Research Center for Children, Youth, & Families, Burlington, VT2003.
214. Pynoos R, Rodriguez J, Steinberg A, Stuber M, Frederick C. UCLA PTSD Index Los Angeles, CA: UCLA; 1998.
215. Pelcovitz D, van der Kolk B, Roth S, Mandel F, Kaplan S, Resick P. Development of a criteria set and a structured interview for disorders of extreme stress (SIDES). J Trauma Stress. 1997;10(1):3-16.
216. Hoff CC, Chakravarty D, Bircher AE, Campbell CK, Grisham K, Neilands TB, et al. Attitudes Towards PrEP and Anticipated Condom Use Among Concordant HIV-Negative and HIV-Discordant Male Couples. AIDS Patient Care STDS. 2015;29(7):408-17.
217. Kalichman SC, Simbayi LC. HIV Testing Attitudes, AIDS Stigma and Voluntary HIV Counseling and Testing in a Black Township in Cape Town, South Africa. Sex Transm Infect. 2003;79(6):442-7.
218. Wilson IB, Lee Y, Michaud J, Fowler FJ, Jr., Rogers WH. Validation of a New Three-Item Self-Report Measure for Medication Adherence. AIDS Behav. 2016;20(11):2700-8.
219. Armsden GC, Greenberg MT. The inventory of parent and peer attachment: Individual differences and their relationship to psychological well-being in adolescence. J Youth Adolesc. 1987;16(5):427-54.
220. Greenberg MT. Personal Communication. 2002.

221. Silvia ES, MacCallum RC. Some factors affecting the success of specification searches in covariance structure modeling. Multivariate Behavioral Research. 1988;23:297-326.
222. Miller KS, Levin ML, Whitaker DJ, Xu X. Patterns of condom use among adolescents: The impact of mother-adolescent communication. Am J Public Health. 1998;88(10):1542-4.
223. Miller KS, Kotchick BA, Dorsey S, Forehand R, Ham AY. Family communication about sex: What are parents saying and are their adolescents listening? Fam Plann Perspect. 1998;30(5):218-22 & 35.
224. Pulerwitz J, Gortmaker SL, DeJong W. Measuring sexual relationship power in HIV/STD research. Sex Roles. 2000;42(7/8):637-60.
225. Sherman SG, Gielen AC, McDonnell KA. Power and attitudes in relationships (PAIR) among a sample low-income, African-American women: Implications for HIV/AIDS prevention. Sex Roles. 2000;42(3/4):283-94.
226. Kalichman SC, Ntseane D, Nthomang K, Segwabe M, Phorano O, Simbayi LC. Recent multiple sexual partners and HIV transmission risks among people living with HIV/AIDS in Botswana. Sex Transm Infect. 2007;83(5):371-5.
227. Harrison A, Cleland J, Frohlich J. Young people's sexual partnerships in KwaZulu-Natal, South Africa: patterns, contextual influences, and HIV risk. Stud Fam Plann. 2008;39(4):295-308.
228. World Health Organization. WHO Multi-country Study on Women’s Health and Life Experiences, Final Core Questionnaire, version 10. Geneva: World Health Organization; 2003.
229. El-Bassel N, Ivanoff A, Schilling RF, Gilbert L, Bourne D, Chen D-R. Preventing HIV/AIDS in drug-abusing incarcerated women through skills-building and social support enhancement: Preliminary outcomes. Soc Work Res. 1995;19(3):131-41.
230. Kalichman SC, Simbayi LC, Jooste S, Toefy Y, Cain D, Cherry C, et al. Development of a Brief Scale to Measure AIDS-Related Stigma in South Africa. AIDS Behav. 2005;9(2):135-43.
231. COVID Exposure and Family Impact Survey: The Center for Pediatric Traumatic Stress; 2020 [Available from: https://www.nlm.nih.gov/dr2/CEFIS_COVID_questionnaire_English_42220_final.pdf.
232. The COVID-19 Questionnaire – Child Self-Report Primary Version, Environmental Influences on Child Health Outcomes (ECHO): Environmental Influences on Child Health Outcomes; 2020 [Available from: https://www.nlm.nih.gov/dr2/C19-cPV_COVID-19_Questionnaire-Child_Self-Report_Primary_Version_20200409_v01.30.pdf.
233. The CoRonavIruS Health Impact Survey (CRISIS) V0.3 Youth Self-Report Baseline Form: Current Form 2020 [Available from: https://www.nlm.nih.gov/dr2/CRISIS_Youth_Self-Report_Baseline_Current_Form_V0.3.pdf.
234. Carroll KC, Aldeen WE, Morrison M, Anderson R, Lee D, Mottice S. Evaluation of the abbott LCx ligase chain reaction assay for detection of chlamydia trachomatis and neisseria gonorrhoeae in urine and genital swab specimens from a sexually transmitted disease clinic population. J Clin Microbiol. 1998;36(6):1630-3.
235. Gaydos C, Howell, M., et al. Use of ligase chain reaction with urine versus cervical culture for detection of Chlamydia trachomatis in an asymptomatic military population of

pregnant and nonpregnant females attending Papnicolaou smear clinics. Journal of Clinical Microbiology,. 1998;36:1300-4.
236. Donenberg GR, Emerson E, Bryant FB, Wilson H, Weber-Shifrin E. Understanding AIDS-risk behavior among adolescents in psychiatric care: Links to psychopathology and peer relationships. Journal of the American Academy of Child Adolescent Psychiatry. 2001;40(6):642-53.
237. Yarnold PR, Bryant FB, Repasy AB, Martin GJ. The factor structure and cross-sectional distributional properties of the Beth Israel/UCLA Functional Status Questionnaire. J Behav Med. 1991;14:141-54.
238. MacCallum RC. Specification searches in covariance structure modeling. Psychological Bulletin,. 1986;100:107-20.
239. Kafka RR, London P. Communication in relationships and adolescent substance use: The influence of parents and friends. Adolescence. 1992;26(103):587-98.
240. Weatherby NL, Needle R, Cesari H. Validity of self-reported drug use among injection drug users and crack cocaine users recruited through street outreach. Education Program Plan. 1994;17:347-55.
241. McKay MM, Baptiste D, McCormick A, Scott R, Paikoff RL, editors. Family based HIV risk exposure prevention programming: A description of the CHAMP family intervention. at the annual meeting at the International Conference on AIDS; 1996; Vancouver, BC.
242. Madison SM, McKay, M.M., Paikoff, R.L., and Bell, C. Basic research and community collaboration: Necessary ingredients for the development of a family-based HIV prevention program. AIDS Educ Prev. 2000;12(4):281-98.
